# *Trypanosoma brucei* TEL2 inhibits VSG switching and protects PIKKs from the 26S proteasome-mediated degradation

**DOI:** 10.1128/spectrum.03516-25

**Published:** 2026-05-29

**Authors:** SK Abdus Sayeed, Prem P. Kushwaha, Marjia Afrin, Bibo Li

**Affiliations:** 1 Center for Gene Regulation in Health and Disease, Department of Biological, Geological, and Environmental Sciences, College of Arts and Sciences, Cleveland State University 2564, Cleveland, Ohio, USA; 2 Case Comprehensive Cancer Center, Case Western Reserve University 2546, Cleveland, Ohio, USA; 3 Department of Inflammation and Immunity, Cleveland Clinic Research, Cleveland, Ohio, USA; 4 Center for RNA Science and Therapeutics, Case Western Reserve University 2546, Cleveland, Ohio, USA; Stony Brook University, Stony Brook, New York, USA; Universidade Estadual Paulista Julio de Mesquita Filho-Campus de Botucatu, Botucatu, Sao Paulo, Brazil

**Keywords:** *Trypanosoma brucei*, TEL2, VSG switching, PI3K-related protein kinases, genome integrity, protein stability

## Abstract

*Trypanosoma brucei* causes debilitating human African trypanosomiasis. It undergoes antigenic variation and sequentially expresses distinct VSGs, its major surface antigen, thereby effectively evading the host’s immune response. We have shown that many telomere proteins suppress VSG switching, as VSGs are expressed exclusively from subtelomeric loci. We have now identified the *T. brucei* TEL2 homolog based on its sequence and predicted structure similarities to those of known TEL2 homologs. TEL2 homologs have a conserved function of maintaining the protein stability of PI3K-related protein kinases (PIKKs). Interestingly, *Tb*TEL2 maintains the stability of *Tb*ATR and most other PIKK proteins by protecting them from the 26S proteasome-mediated degradation. This is different from mechanisms revealed in mammals and fission yeast, where TEL2 homologs act as an HSP90 co-chaperone and ensure co-translational maturation of PIKKs. In addition, *Tb*TEL2 is essential for genome integrity and acts as another critical player in antigenic variation regulation. More DNA breaks have been detected at multiple *T. brucei* loci, including both active and silent subtelomeric VSG expression sites in *Tb*TEL2-depleted cells. Furthermore, a transient depletion of *Tb*TEL2 leads to a significant increase in the number of VSG switching events via DNA recombination-mediated and transcriptional switching pathways. Hence, the increased genome instability promotes antigenic variation potential. However, only a trace amount of γH2A, a marker of DNA damage, was detected transiently upon *Tb*TEL2 depletion, presumably due to the diminution of H2A kinases in these cells, suggesting that persistent ATM/ATR-mediated damage signaling is not essential for the increase in VSG switching rate.

**IMPORTANCE:**

*Trypanosoma brucei* causes sleeping sickness in humans and nagana in cattle, causing a severe economic burden in sub-Sahara Africa. Antigenic variation is a key pathogenesis mechanism that enables long-term parasitic infections and renders vaccination ineffective. We have identified *T. brucei* TEL2 and shown that it is indispensable for parasite proliferation, essential for genome integrity, and critical for the regulation of antigenic variation. Importantly, *Tb*TEL2 maintains the stability of PIKK proteins that are key players in various important cellular processes, indicating that *Tb*TEL2 is a central regulator essential for parasite survival in its mammalian host. Importantly, we found that *Tb*TEL2 protects PIKKs from the 26S proteasome-mediated protein degradation. This is distinct from the scenarios in mammals and fission yeast, where TEL2 homologs act as an HSP90 co-chaperone and ensure co-translational maturation of PIKKs. Characterizing the parasite’s essential processes with unique mechanisms will help eventual eradication of *T. brucei* infections.

## INTRODUCTION

*Trypanosoma brucei* causes human African trypanosomiasis, which is frequently fatal if left untreated. While proliferating in the extracellular spaces of its mammalian host, *T. brucei* sequentially expresses distinct variant surface glycoproteins (VSGs), its major surface antigen, thereby effectively evading the host’s immune response. All *VSG* genes and pseudogenes are located at subtelomeres (1–3), and VSGs are expressed exclusively from subtelomeric VSG expression sites (ESs) in a strictly monoallelic manner (4, 5).

Antigenic variation is a key pathogenic mechanism that enables the parasite to establish a long-term infection. VSG monoallelic expression mainly relies on *Tb*RAP1, a telomere chromatin-associated protein (6–12); PIP5Pase, which is involved in the inositol phosphate pathway and interacts with *Tb*RAP1 (13–16); VEX1/VEX2, which associate with the active *VSG* ESs (17–20); and ESB1 and ESBX, which associate with DNA near the active ES promoter and are required for the active *VSG* expression and silent *VSG* exclusion (21, 22). In addition, cells with extremely short telomeres have a higher VSG switching rate, with gene conversion being the more prevalent switching mechanism (23). Our lab has also demonstrated that many *T. brucei* telomere proteins, including *Tb*TRF, *Tb*RAP1, *Tb*TIF2, and PolIE, not only are essential for genome integrity but also suppress DNA recombination-mediated VSG switching (8, 24–32).

As telomeres play important roles in antigenic variation, we explored functions of a potential telomere component. In a recent Proteomics of Isolated Chromatin segment (PICh) experiment, we identified *Tb*927.5.670 as a protein that potentially associates weakly with the telomere chromatin (30). Interestingly, we have identified this protein as a TEL2 homolog independently based on its sequence homology with other TEL2 homologs. The *Saccharomyces cerevisiae* TEL2 was first identified based on the observation that the *tel2-1* mutant has shorter telomeres (33). TEL2 homologs were later identified in mammals (34–36), fission yeast (37), plants (38), and worms (39, 40) and were initially shown to be central players in the DNA damage response (35–37, 41), but were never identified as core components of the telomere complex. Interestingly, TEL2 homologs have been shown to regulate protein levels of PI3K-related protein kinases (PIKKs) (42), which are central regulators of key cellular processes, including growth control, cell survival, metabolic regulation, and immune responses (43). Mammalian PIKKs have six members: ATM, ATR, DNA-PK, mTOR, SMG1, and TRRAP (44), while fission yeast PIKKs include Tel1, Rad3, Tor1, Tor2, Tra1, and Tra2 (45, 46). Mammalian and fission yeast TEL2s interact with all six PIKKs (36, 46), and budding yeast TEL2 interacts with Tel1 (ATM homolog) and Mec1 (ATR homolog) (41). Although TEL2 regulates PIKK protein levels post-translationally, mammalian TEL2 does not protect PIKKs from proteasome-mediated degradation, as adding a proteasome inhibitor does not rescue the loss of PIKK proteins in TEL2-deleted cells (36). Instead, in mammals and yeasts, the TEL2-TTI1-TTI2 heterotrimer interacts with the Rvb1-Rvb2-Tah1-Pih1 (R2TP) complex (47) and acts as a co-chaperone of HSP90 to ensure co-translational maturation of PIKKs (41, 48–52).

In *T. brucei*, six PIKKs have been identified: *Tb*ATM (*Tb*927.2.2260), *Tb*ATR (*Tb*927.11.14680), *Tb*TOR1 (*Tb*927.10.8420), *Tb*TOR2 (*Tb*927.4.420), *Tb*TOR3 (*Tb*927.4.800), and *Tb*TOR4 (*Tb*927.1.1930). *Tb*ATM and *Tb*ATR are major players in DNA damage response pathways (53, 54), with *Tb*ATM being the primary kinase that phosphorylates H2A in response to ionizing irradiation (53), while *Tb*ATR has been shown to play a role in the regulation of antigenic variation (55). *Tb*TOR1 regulates protein synthesis and cell size and controls autophagy (56, 57); *Tb*TOR2 plays an important role in cytokinesis (56, 58); and *Tb*TOR3 (also known as *Tb*TOR-like 1) controls polyphosphate levels and acidocalcisome maintenance (59). Finally, depletion of *Tb*TOR4 pre-adapts bloodstream-form *T. brucei* cells for differentiation into the insect stage (60). Therefore, like PIKK proteins in other organisms, *T. brucei* PIKKs also play important roles in growth regulation and cell survival. However, whether *Tb*TEL2 has any functions in antigenic variation or regulates PIKK protein levels was unknown.

In this study, we found that *Tb*TEL2 is essential for cell proliferation. *Tb*TEL2 is required for strict VSG monoallelic expression and suppresses VSG switching, although depletion of *Tb*TEL2 or expression of an ectopic *TbTEL2* allele does not cause dramatic changes in the telomere growth rate. In addition, *Tb*TEL2 is essential for maintaining genome integrity. Like other TEL2 homologs, *Tb*TEL2 helps maintain the protein stability of most PIKKs. We also find that *Tb*TEL2 interacts with *Tb*ATR. Surprisingly, different from mammalian and fission yeast TEL2 homologs, *Tb*TEL2 inhibits proteasome-mediated protein degradation of *Tb*ATR, *Tb*TOR1, T*b*TOR2, and *Tb*TOR4, revealing conserved TEL2 functions with different underlying mechanisms in different organisms. In addition, only a weak γH2A signal has been transiently detected in *Tb*TEL2-depleted cells, presumably due to lower *Tb*ATM/*Tb*ATR levels in these cells, suggesting that *Tb*ATM/*Tb*ATR-independent DNA damage response pathway(s) may help induce more VSG switching events, or persistent damage response signaling may not be required for initiating VSG switching.

## RESULTS

### Identification of the *T. brucei* TEL2 homolog

**Fig 1:**
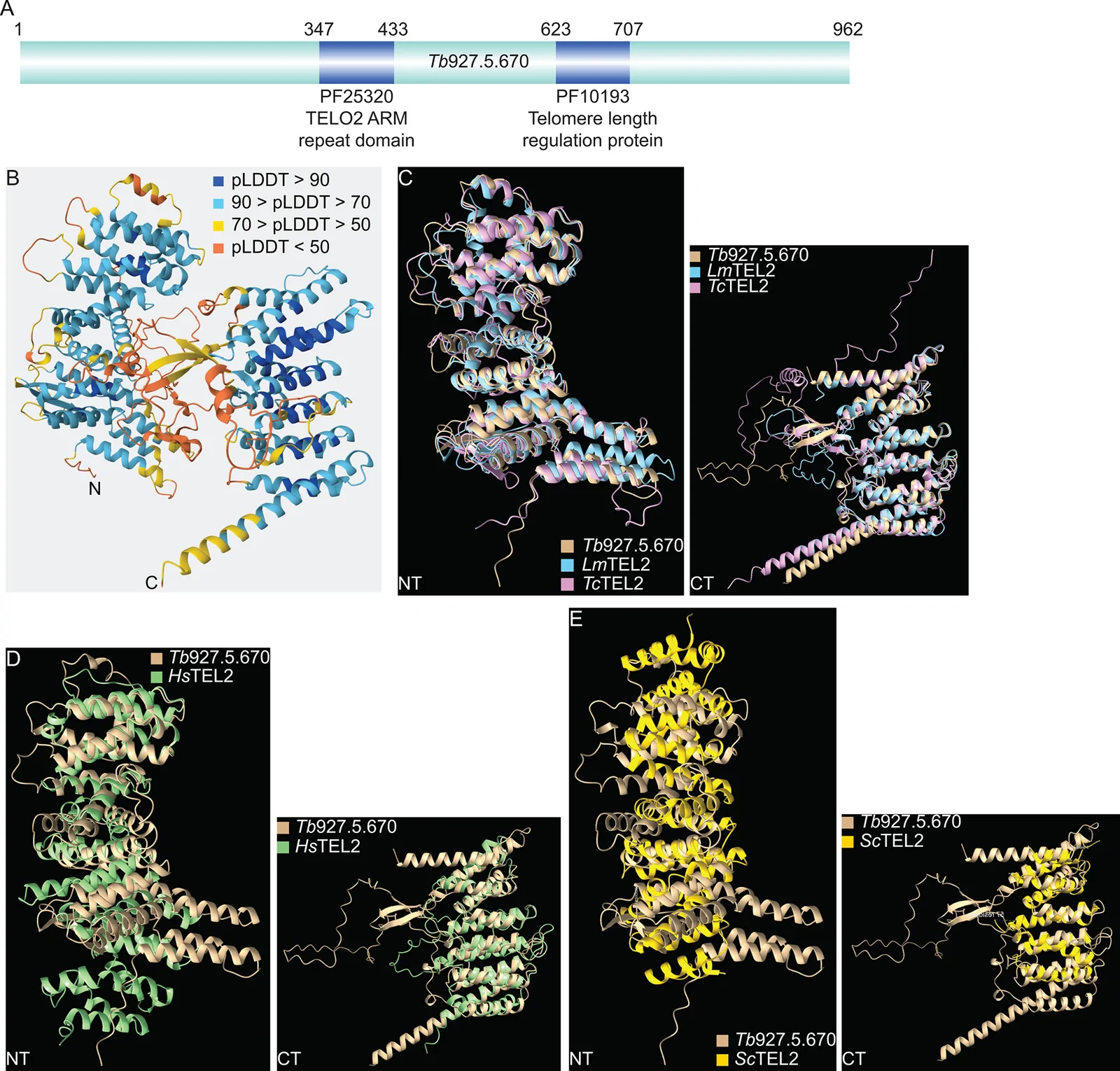
*Tb*927.5.670 is a TEL2 homolog. (**A**) Domain structure of *Tb*927.5.670. PF25320, a predicted ARM-type repeat region in TEL2 homologs. (ARM repeats are α-helical repeat motifs that form elongated, superhelical solenoids specialized for protein–protein interactions.) PF10193, a conserved, TEL2-specific structural domain required for the TEL2-TTI1-TTI2 (TTT) scaffold that is needed for PIKK maturation. (**B**) AlphaFold 3-predicted protein structure of *Tb*927.5.670, which has an N-terminal (NT, left) and a C-terminal (CT, right) portion connected by flexible loops (middle). pLDDT: a per-atom confidence estimate on a 0–100 scale, where a higher value indicates higher confidence (61, 62). (**C**) Alignments of AlphaFold 3-predicted structures of *Tb*TEL2, *Lm*TEL2, and *Tc*TEL2 NT (left) and CT (right) halves using ChimeraX. (**D**) Alignments of AlphaFold 3-predicted structures of *Tb*TEL2 and *Hs*TEL2 NT (left) and CT (right) halves using ChimeraX. (**E**) Alignments of *Tb*TEL2 NT (left) and CT (right) structures (AlphaFold 3-predicted) and corresponding regions of *Sc*TEL2 (solved X-ray structure, PDB ID: 3O4Z [51]) using ChimeraX. Additional alignment parameters for panels **C–E** are listed in Table S2.

TEL2 homologs have been identified in many organisms, including *Leishmania major* (39), although the function of *Lm*TEL2 has not been characterized. Since *Leishmania* and *T. brucei* are closely related kinetoplastids, we used *Lm*TEL2 (*Lm*jF.35.0870) as a query for a BLASTP search in the *T. brucei* TREU927 genome and identified *Tb*927.5.670 with a score of 392 and an e-value of 1E-121. Sequence alignment using ClustalW (https://www.genome.jp/tools-bin/clustalw) shows that *Tb*927.5.670 has notable sequence homology to other TEL2 homologs and is most closely related to *T. cruzi* and *Leishmania* TEL2 homologs (Fig. S1A). Motif Search (https://www.genome.jp/tools/motif/) analysis indicates that *Tb*TEL2 has two Pfams that are found in TEL2 homologs (Fig. 1A). In addition, AlphaFold 3 (61, 62) predicts that *Tb*927.5.670 has a structure containing two α-solenoids—flexible protein structural domains formed by ensembles of alpha-helical repeats (63)—at the N- and C-terminal halves of the protein (NT and CT, Fig. 1B), which is similar to the solved *Sc*TEL2 X-ray structure (PDB ID: 3O4Z) (51) and the predicted structures of other TEL2 homologs. However, since the inter-domain arrangements are flexible, full-length (FL) TEL2 structures do not align well. Therefore, we examined the structures of NT and CT of known TEL2 homologs separately. For all TEL2 homologs, the predicted structures are nearly identical regardless of whether FL or NT/CT sequences were used (e.g., alignments of FL and NT/CT structures for *Tb*927.5.670 and *Hs*TEL2 in Fig. S1B and C). Importantly, the *Sc*TEL2 structure (PDB ID: 3O4Z) (51) and predicted NT and CT structures of other TEL2 homologs all align reasonably well with *Tb*927.5.670 (Fig. S1D and Table S1), suggesting good structure similarities among all TEL2 homologs. The kinetoplastid TEL2 homologs (*Tb*927.5.670, *Lm*TEL2, and *Tc*TEL2) are clearly more closely related (Fig. S1A, inset), and their predicted structures are well-conserved (Fig. 1C and Table S2). *Tb*927.5.670 also aligns well to *Hs*TEL2 and *Sc*TEL2 (Fig. 1D and E; Table S2). These observations strongly suggest that *Tb*927.5.670 is a TEL2 homolog, and we name it *Tb*TEL2.

### TbTEL2 is essential for *T. brucei* proliferation, but perturbation of the TbTEL2 expression level does not cause dramatic changes in the telomere growth rate

**Fig 2:**
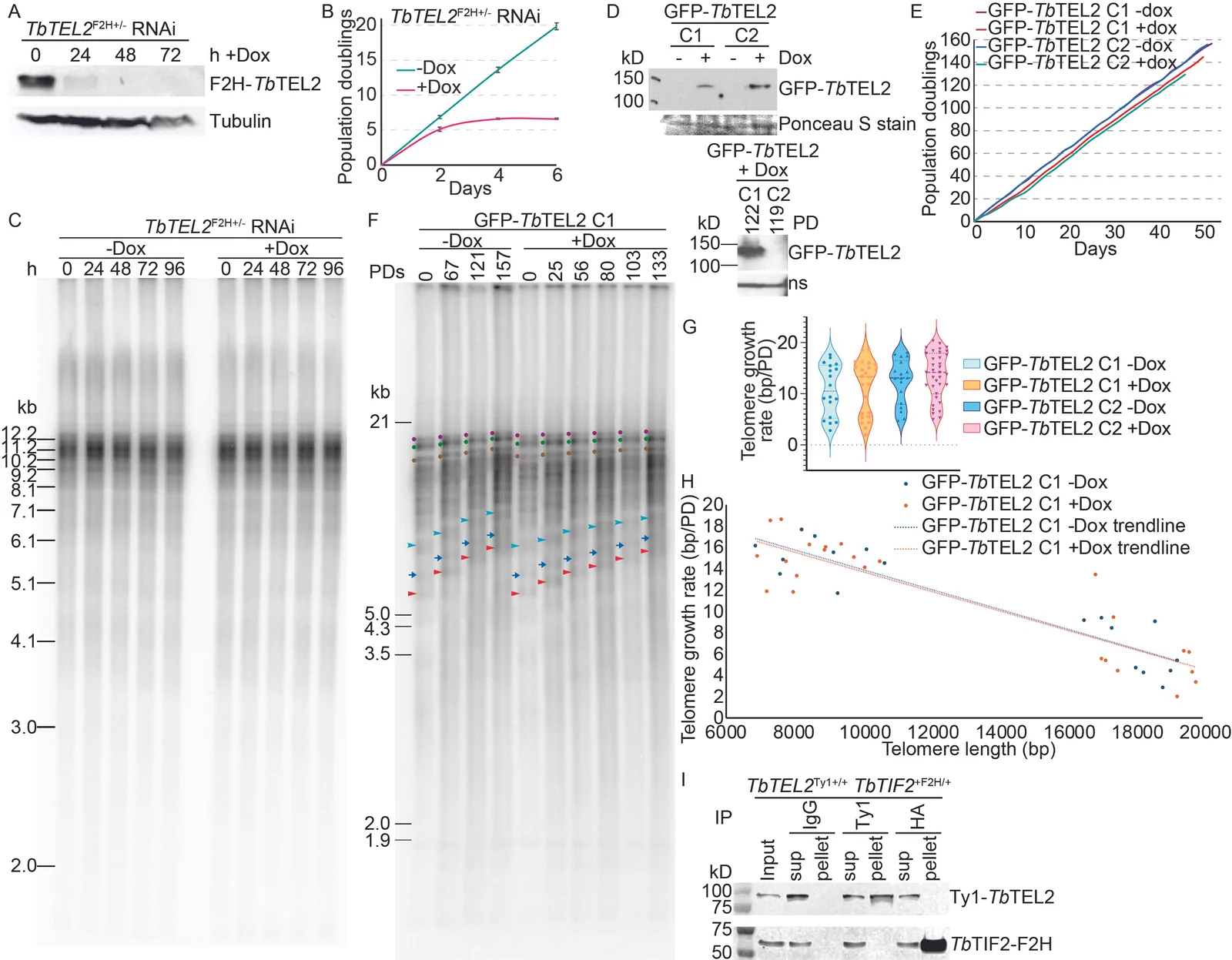
*Tb*TEL2 is essential for *T. brucei* proliferation. (**A**) Western blotting using an HA monoclonal antibody (HA-probe, Santa Cruz Biotechnology) shows the depletion of F2H-*Tb*TEL2 in *TbTEL2*^F2H+/−^ RNAi cells upon adding 100 ng/mL doxycycline. (**B**) Growth curves of *TbTEL2*^F2H+/−^ RNAi cells with and without doxycycline. Error bars represent standard deviation. (**C–H**) Depletion of *Tb*TEL2 or expression of an ectopic *TbTEL2* allele does not cause dramatic changes in telomere growth rate. (**C**) *TbTEL2*^F2H+/−^ RNAi cells were cultured with or without doxycycline for 4 days, and genomic DNAs were isolated every 24 h. Genomic DNA was digested by AluI and MboI followed by in-gel hybridization using an end-labeled TELC4 oligo probe. (**D**) Western blotting using a rabbit anti-GFP antibody (Thermo Fisher Scientific) to detect the expression of GFP-*Tb*TEL2 at 24 h (top) and ~120 PDs (bottom) after induction. As a loading control, Ponceau S stain of a protein band at ~75 kDa (top) and a non-specific protein band (bottom) are shown. Two independent clones were examined. (**E**) Growth curves of C1 and C2 cells under induced and uninduced conditions. (**F**) GFP-*Tb*TEL2 C1 cells with or without induction were cultured for ~50 days, and genomic DNAs were isolated at various time points (as indicated on top of the gel). Genomic DNAs were digested by AluI and MboI followed by Southern blotting using an 800 bp TTAGGG repeat probe. The sizes of six telomere fragments were quantified (marked by red, blue, and cyan arrow/arrow heads and purple, green, and brown dots). (**G**) Summary of telomere growth rates in induced and uninduced C1 and C2 cells. (**H**) Telomere growth rate vs telomere band size is plotted for induced and uninduced C1 cells. The trend lines for both groups of data are shown as dotted lines. (**I**) *Tb*TEL2 does not interact with *Tb*TIF2. In *TbTEL2*^Ty1+/+^ *TbTIF2*^+F2H/+^ cells, co-IP experiments were performed using a mouse anti-Ty1 antibody, a mouse anti-HA antibody, and normal mouse IgG (as a negative control), and the IP products were detected by Western blotting using a rabbit anti-Ty1 antibody (top) and a rabbit anti-HA antibody (bottom).

To examine *Tb*TEL2’s functions, we first replaced one *TbTEL2* allele with the blasticidin S resistance marker (*BSD*) and inserted an N-terminal FLAG-HA-HA (F2H) tag into the other *TbTEL2* endogenous allele, allowing us to detect F2H-*Tb*TEL2 with HA antibodies and subsequently facilitating efficient *Tb*TEL2 depletion by RNAi. The genotype of the resulting *TbTEL2*^F2H+/−^ strain (Table S3) was validated by PCR (Fig. S2A and B), and expression of F2H-*Tb*TEL2 was confirmed by Western blotting (Fig. S2C). Subsequently, we targeted a *Tb*TEL2 RNAi construct to an rDNA spacer in *TbTEL2*^F2H+/−^ cells to establish the *TbTEL2*^F2H+/−^ RNAi strain (Table S3). Upon induction of *Tb*TEL2 RNAi, most of the F2H-*Tb*TEL2 protein was depleted by 24 h, and no protein was detectable by 48 h (Fig. 2A). Consequently, we also observed that the *TbTEL2*^F2H+/−^ RNAi cells experienced a growth arrest upon induction of *Tb*TEL2 RNAi (Fig. 2B), indicating that *Tb*TEL2 is essential for normal cell proliferation. Importantly, *TbTEL2*^F2H+/−^, *TbTEL2*^+/−^, and *TbTEL2*^+/+^ cells grow nearly identically (Fig. S2D), indicating that F2H-*Tb*TEL2 retains *Tb*TEL2’s essential functions.

We were curious whether *Tb*TEL2, like *Sc*TEL2, is involved in telomere length regulation. First, the *TbTEL2*^F2H+/−^ RNAi cells were induced for 4 days before indirect effects may arise due to long-term cell growth arrest, and telomere length changes were examined by Southern in-gel hybridization. In *TbTEL2*^F2H+/−^ RNAi cells, we did not detect any discernible telomere length changes with or without inducing *Tb*TEL2 RNAi (Fig. 2C). Since the uninduced cells had only grown for 13.2 population doublings (PDs) and the induced cells for 6.4 PDs, and *T. brucei* telomeres normally elongate at a rate of 9–15 bp/PD (64–66), the maximal telomere elongation mediated by telomerase in these cells would be ~200 bp after 4 days of growth. The bulk telomeres in *TbTEL2*^F2H+/−^ RNAi cells are between 8 and 20 kb long (Fig. 2C). Therefore, Southern in-gel hybridization is not sensitive enough to detect small telomere length changes within this short time frame. Importantly, we did not observe any dramatic telomere length changes that are frequently mediated by telomere recombination (67–69), suggesting that *Tb*TEL2 depletion does not activate a telomerase-independent alternative telomere maintenance mechanism that usually depends on telomere recombination, at least within a few days.

It is well known that *T. brucei* telomeres continue to grow during cell proliferation with an average elongation rate of 9–15 bp/PD, which mainly depends on telomerase (64–66, 70). The active *VSG*-adjacent telomere has a much more dynamic size range (64, 71). In addition, adding back telomerase to telomerase-null cells with short telomeres and telomere healing experiments showed that the newly formed active telomere has a dramatically faster elongation rate than silent telomeres (72, 73). Telomere healing experiments also imply that shorter telomeres are elongated at a faster rate than longer telomeres (73). However, a careful comparison of growth rates between short and long telomeres among all telomeres has not been reported. In WT *T. brucei* cells, we performed Southern hybridization to examine the bulk telomere length changes. As expected, all telomeres continue to grow as the cells proliferate (Fig. S3A). We quantified the size changes of many prominent telomere bands (Fig. S3A, marked telomere bands) and plotted telomere growth rate vs telomere fragment size (Fig. S3B). Linear regression analysis indicates that the slope is −0.0012 (Fig. S3B) with a two-sided slope test yielding a *P* value of 5.5E-16. Pearson correlation and Spearman rank correlation tests both indicate that there is a strong, highly significant negative association between telomere size and telomere growth rate, with *P* values of 5.5E-16 and 3.0E-16, respectively.

*Tb*TEL2 depletion leads to an acute growth arrest (Fig. 2B), preventing us from examining telomere length changes in an extended period. Therefore, we determined whether expressing an ectopic WT *TbTEL2* allele affects telomere length in the long term. A conditional inducible GFP-*Tb*TEL2 expression construct was inserted at an rDNA spacer to generate two independent clones of GFP-*Tb*TEL2 (Table S3). Adding doxycycline to these cells led to expression of ectopic GFP-*Tb*TEL2 (Fig. 2D, top). Inducing GFP-*Tb*TEL2 expression also caused a transient growth delay in the first 3 days, but cells recovered and grew at the same speed as uninduced cells subsequently (Fig. 2E). We cultured the induced and uninduced GFP-*Tb*TEL2 cells (both clones) for more than 50 days. After ~120 PDs of cell growth, Western blotting showed that C1 cells still expressed GFP-*Tb*TEL2, but C2 did not (Fig. 2D, bottom). We examined the telomere length changes in both clones by Southern blotting using a telomere probe (Fig. 2F and Fig. S3C), where C2 cells were used as a negative control. We quantified the telomere growth rates following several distinct telomere bands (Fig. 2F and Fig. S3C, marked bands) and found that the telomere growth rates have a large distribution for both clones (Fig. 2G). When telomere fragment size was plotted against telomere growth rate, we again observed an inverse relationship of the two factors (Fig. 2H and Fig. S3D). Importantly, ANCOVA analyses using models with and without interaction both indicate that expression of ectopic GFP-*Tb*TEL2 does not cause significant changes in telomere growth rates. For C1 cells, the two groups of data (with and without inducing GFP-*Tb*TEL2 expression) have parallel slopes (−0.0009), and the interaction term is non-significant (*P* = 0.91) (Fig. 2H). For C2 cells, the two groups of data (in the presence and absence of Dox) have similar slopes (−0.0009 and −0.0011, respectively), and the interaction term is non-significant (*P* = 0.17) (Fig. S3D). Therefore, regardless of whether the ectopic GFP-*Tb*TEL2 is expressed for an extended period, telomere growth rate is not affected. Nevertheless, our data further verified that shorter telomere fragments elongate faster than longer ones in *T. brucei*.

Interestingly, in a Proteomics of Isolated Chromatin segment (PICh) experiment, we pulled down the *T. brucei* telomere chromatin, and *Tb*TEL2 was identified as a protein potentially weakly associated with telomeric chromatin (30). Therefore, we explored whether *Tb*TEL2 interacts with key telomere proteins in *TbTEL2*^F2H+/+^ cells (Table S3). *Tb*RAP1 is a telomere protein that plays critical roles in antigenic variation (6–11). However, we did not detect *Tb*RAP1 in the *Tb*TEL2 IP product (Fig. S4A). *Tb*TIF2 is a telomere protein that plays an essential function in telomere integrity (25, 26). To examine potential interaction between *Tb*TEL2 and *Tb*TIF2, we established a strain in which one endogenous *TbTEL2* allele has an N-terminal Ty1 tag (Fig. S4B and C) and one endogenous *TbTIF2* allele has a C-terminal F2H tag (Fig. S4D and E). However, we did not detect *Tb*TEL2 and *Tb*TIF2 simultaneously in their respective IP products (Fig. 2I). Since *Tb*TIF2 and *Tb*TRF—a major duplex telomeric DNA binding factor in *T. brucei* (24, 27, 28)—form a tight protein complex (26), this observation suggests that *Tb*TEL2 does not interact with *Tb*TRF either. Furthermore, we did not observe any *Tb*TRF protein level change upon *Tb*TEL2 depletion (Fig. S4F). Finally, the tryptag.org database shows that N-terminal or C-terminal mNeoGreen-tagged *Tb*TEL2 is mostly located in the cytoplasm (74). Therefore, like other TEL2 homologs, *Tb*TEL2 is not a core component of the telomere complex.

### TbTEL2 suppresses VSG switching by maintaining genome integrity

**Fig 3:**
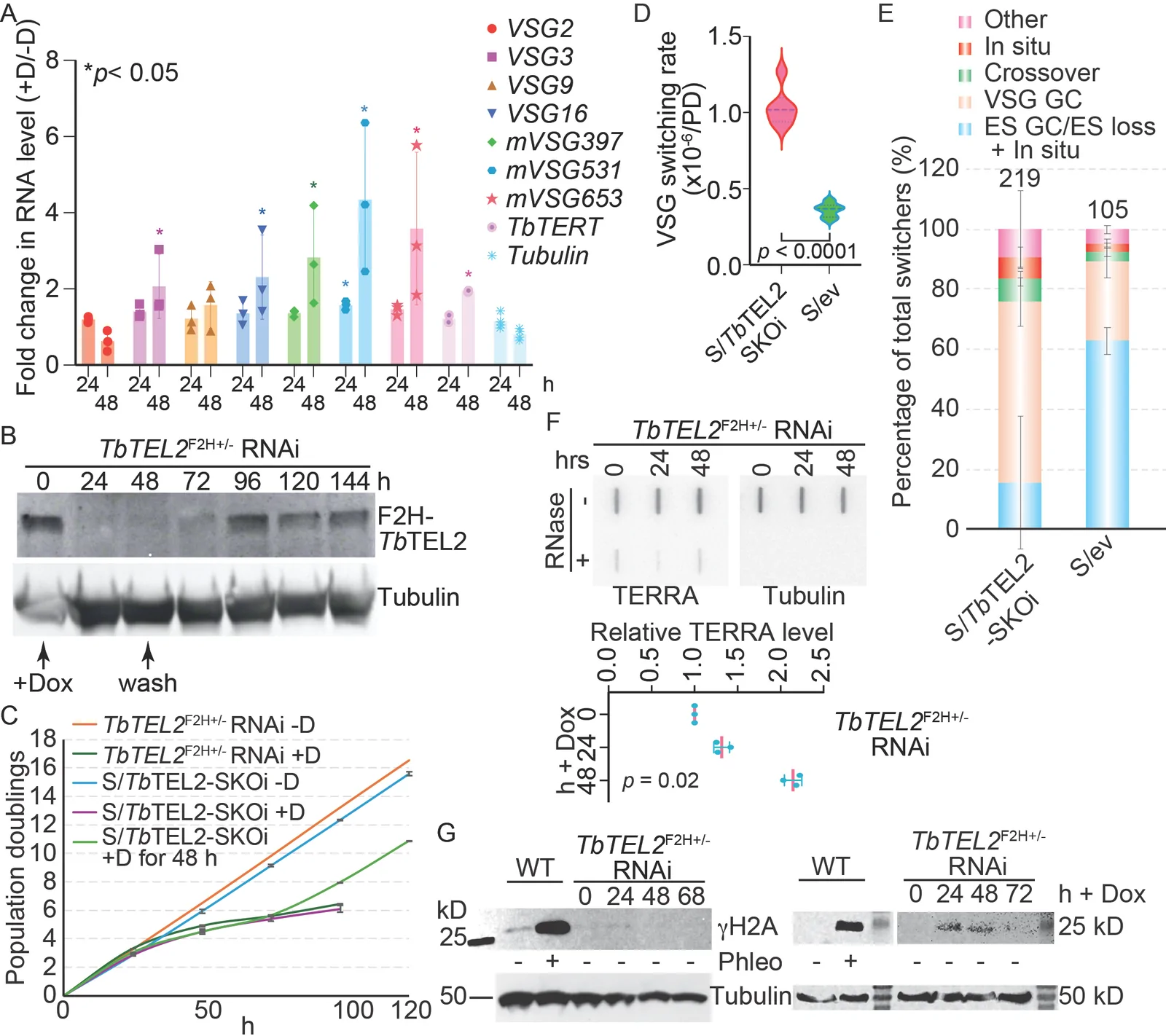
*Tb*TEL2 suppresses VSG switching by maintaining genome integrity. (**A**) *Tb*TEL2 depletion leads to mild derepression of selected silent *VSG* genes. Total RNA was isolated from *TbTEL2*^F2H+/−^ RNAi cells at 0, 24, and 48 h after adding doxycycline. qRT-PCR was performed to examine the RNA levels of selected *VSG* genes. (**B–E**) A transient depletion of *Tb*TEL2 leads to an increased VSG switching rate. (**B**) Western blotting to examine the F2H-*Tb*TEL2 level using the HA probe antibody (Santa Cruz Biotechnology) (top) in *TbTEL2*^F2H+/−^ RNAi cells that were induced for 48 h. Tubulin was detected by TAT-1 (75) as a loading control (bottom). (**C**) Growth curves of *TbTEL2*^F2H+/−^ RNAi cells cultured in the presence and absence of doxycycline and those of S/*Tb*TEL2-SKOi cells cultured in the presence (for 96 h or 48 h) and absence of doxycycline. (**D**) VSG switching rates in S/*Tb*TEL2-SKOi cells induced for 48 h and in S/ev control cells. Average VSG switching rate was calculated from six groups of obtained switchers. (**E**) Percentage of various VSG switching mechanisms among all switchers (total number of analyzed switchers is indicated on top of each column). (**F**) Depletion of *Tb*TEL2 leads to an increase in the TERRA level. A representative TERRA Northern slot blot is shown at the top, and the quantification of three slot blots is shown at the bottom. (**G**) Western blotting using a rabbit antibody specific for γH2A (top) (8). Whole-cell lysates were isolated from *TbTEL2*^F2H+/−^ RNAi cells at various time points after induction (indicated on the top). As a positive control, WT cells were treated with phleomycin for 24 h, and whole-cell lysates were isolated before and after phleomycin treatment. Tubulin was detected as a loading control (bottom). Two independent experiment results are shown. The right γH2A Western image was exposed for a longer time. Error bars in panels **A**, **C**, **E**, and **F** represent standard deviation.

To examine *Tb*TEL2’s role in antigenic variation, we performed qRT-PCR to examine RNA levels of several *VSGs* in bloodstream-form VSG expression sites (BESs) or metacyclic VSG expression sites (MESs) in *TbTEL2*^F2H+/−^ RNAi cells before and after inducing *Tb*TEL2 RNAi (at 0, 24, and 48 h post-induction). The active *VSG2* RNA level was not significantly affected (Fig. 3A and Table S4). BES-linked *VSG3* and *VSG16* were mildly derepressed (~2-fold) upon depletion of *Tb*TEL2, while MES-linked *mVSG397*, *mVSG531*, and *mVSG635* were derepressed 3- to 5-fold (Fig. 3A and Table S4), indicating that *Tb*TEL2 is required to maintain strict VSG monoallelic expression, although the effect of *Tb*TEL2 depletion on *VSG* silencing is mild.

To accurately measure the VSG switching rate, we used the HSTB261 strain (named “S” for simplicity, standing for switching), which has a *BSD* marker immediately downstream of the active BES promoter and a *PUR-TK* marker immediately upstream of the active *VSG2* (Fig. S5A, Parent) (76). Selecting these cells with blasticidin and puromycin before the commencement of the VSG switching assay ensures that the starting cell population uniformly expresses VSG2. We replaced one endogenous *TbTEL2* allele with the hygromycin resistance marker (*HYG*) and transfected the *Tb*TEL2 RNAi construct in these cells to establish the S/*Tb*TEL2-SKOi strain (Table S3, Fig. S5B and C). Since depletion of *Tb*TEL2 leads to a growth arrest, we first tested the transient *Tb*TEL2 depletion condition for switcher recovery. The *TbTEL2*^F2H+/−^ RNAi cells were induced for 48 h before the doxycycline was removed (by extensive washing). We observed efficient depletion of *Tb*TEL2 upon induction and prompt recovery of the *Tb*TEL2 protein level after doxycycline removal (Fig. 3B). S/*Tb*TEL2-SKOi and *TbTEL2*^F2H+/−^ RNAi cells grew similarly upon induction of *Tb*TEL2 RNAi (Fig. 3C). Importantly, the S/*Tb*TEL2-SKOi cells recovered their normal growth after a short-term growth retardation due to the transient *Tb*TEL2 depletion (Fig. 3C), confirming that we can examine the effect of transient *Tb*TEL2 depletion on VSG switching. To perform the VSG switching assay, S/*Tb*TEL2-SKOi cells were first washed extensively to remove blasticidin and puromycin to allow switchers to arise (8, 76). S/*Tb*TEL2-SKOi cells were induced for 48 h before doxycycline was removed by extensive washing, and both S/*Tb*TEL2-SKOi and the control S/ev cells (Table S3) were cultured for a total of ~10.5 PDs. Switchers that no longer express the originally active VSG2 (validated by Western slot blot using a VSG2-specific antibody) or TK were selected by ganciclovir (GCV) and further validated by their sensitivity to puromycin. This way all switchers, regardless of which VSG is expressed after switching, can be obtained. Switchers’ sensitivity to BSD was tested, and whether the switchers retain intact *VSG2* and *BSD* genes was examined by PCR. Based on the collective phenotype and genotype pattern, we determined the switching mechanism for each switcher (Fig. S5A and Table S5) (8, 76). Quantification and characterization of obtained VSG switchers showed that a transient depletion of *Tb*TEL2 led to an approximate 3-fold increase in the VSG switching rate (Fig. 3D). Comparing distributions of various VSG switching mechanisms in *Tb*TEL2-depleted cells and S/ev cells, we observed increased fractions of *VSG* gene conversion, reciprocal crossover, *in situ*, and other switching events, but a decreased fraction of ES gene conversion/ES loss + *in* situ events upon *Tb*TEL2 depletion (Fig. 3E and Table S5). An exact Monte Carlo test (Fisher–Freeman–Halton type) showed that this difference was significant (*P* < 0.00001). Therefore, *Tb*TEL2 suppresses VSG switching.

We previously showed that an increased level of the telomere transcript (TERRA) can lead to an elevated VSG switching rate (8, 28, 77). Therefore, we further examined whether *Tb*TEL2 depletion leads to an increased TERRA level. Total RNA was isolated, and the TERRA level was estimated by Northern slot blot hybridization using a telomere probe and a tubulin probe (as a loading control). Interestingly, depletion of *Tb*TEL2 resulted in a 2-fold increase in the TERRA level (Fig. 3F). A higher level of TERRA can form more telomeric R-loops (TRLs), which frequently lead to more telomeric/subtelomeric DNA damage and subsequent elevated VSG switching rate (8, 28). Therefore, we also examined the level of γH2A in *TbTEL2*^F2H+/−^ RNAi cells, where γH2A has been shown to be a marker for DNA damage in *T. brucei* (78). As a positive control, treating WT cells with phleomycin induced a dramatic increase in the γH2A signal as expected (Fig. 3G). However, *Tb*TEL2 depletion only led to a short-term increase in the γH2A signal. Weak signals of γH2A were detected 24 h and 48 h after *Tb*TEL2 RNAi induction, which were lost by 72 h after induction (Fig. 3G). In addition, Western blotting showed that *Tb*RAD51’s protein level increased at 24 and 48 h but started to drop at 72 h after *Tb*TEL2 RNAi induction (Fig. S5D).

**Fig 4:**
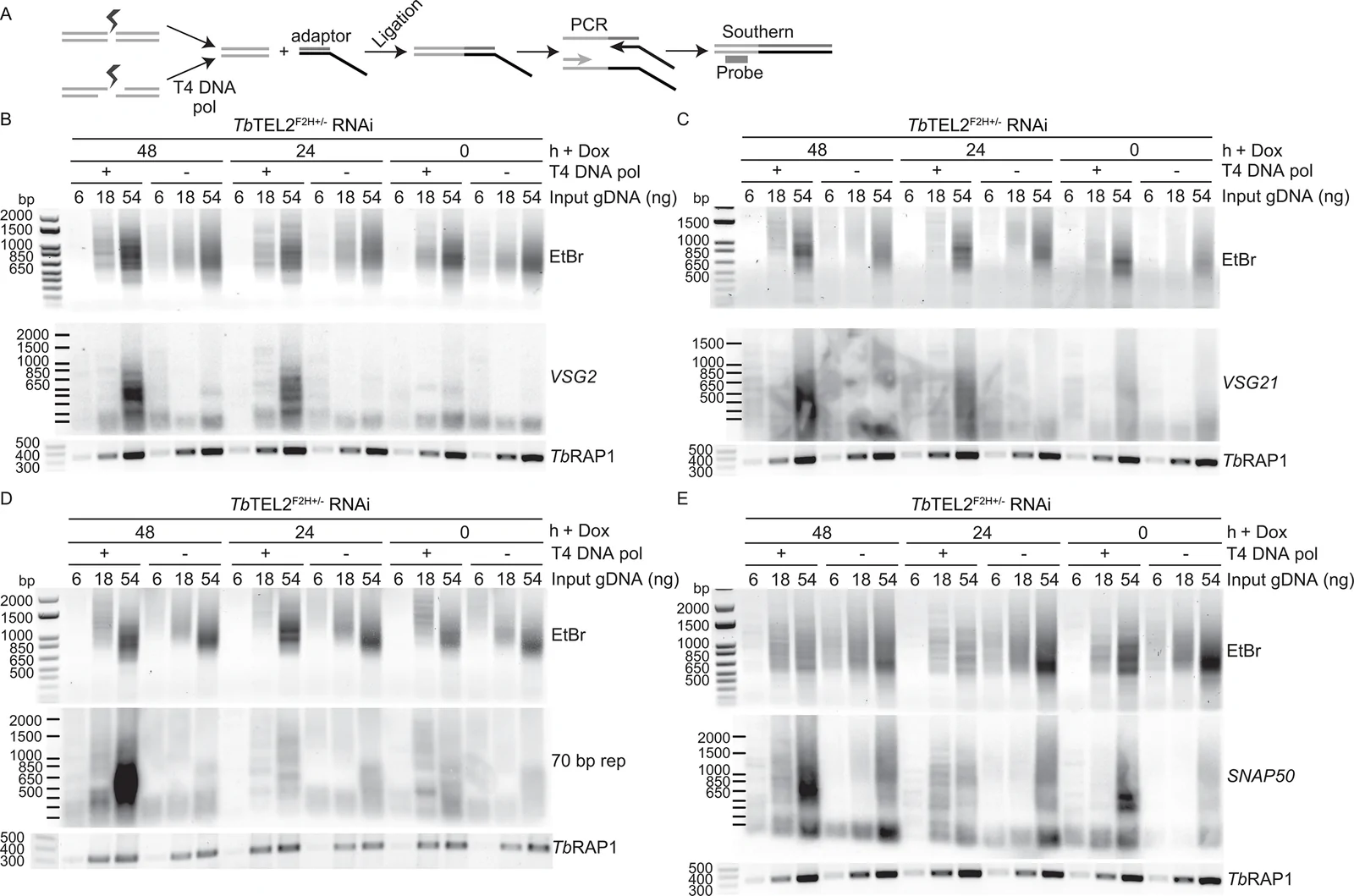
Depletion of *Tb*TEL2 leads to more DNA breaks. (**A**) A diagram depicting the LMPCR principle. Locus-specific primers at the active *VSG2* (**B**), silent *VSG21* (**C**), 70 bp repeats (**D**), and chromosome-internal *SNAP50* (**E**) were used in LMPCR. The amplified ligated products were detected by corresponding locus-specific probes in Southern hybridization analyses. In each panel, the EtBr-stained gel (top), the hybridization result (middle), and a PCR amplified *Tb*RAP1 fragment (as a loading control, bottom) are shown. Panels **B, C**, and **E** were done using exactly the same genomic DNA sample, and the same loading controls are shown.

It has been shown that DNA breaks, particularly those at the active *VSG* locus, can induce VSG switching efficiently (79, 80). Our previous observations also indicate that elevated VSG switching rate can be induced by DNA breaks at the telomere/subtelomere vicinity (8, 26, 28). To test whether *Tb*TEL2 depletion leads to more DNA breaks, we performed ligation-mediated PCR (LMPCR) at selected loci of *T. brucei* genome. LMPCR can detect DNA breaks directly regardless of whether a DNA damage response pathway is activated. In LMPCR, DNA breaks with blunt ends can directly ligate to an adaptor having one blunt end and one cohesive end with a single-stranded 5′ overhang. Cohesive DNA break ends can be converted to blunt ends by T4 DNA polymerase and then ligated with an adaptor. The ligated products can be amplified by PCR using locus-specific primers and detected by Southern hybridization using locus specific probes (Fig. 4A). In *TbTEL2*^F2H+/−^ RNAi cells, we detected DNA breaks even without the T4 DNA polymerase treatment, indicating that some DNA breaks already had blunt ends (Fig. 4B through E). Many more DNA breaks were detected by LMPCR after genomic DNA was treated with T4 DNA polymerase, indicating that abundant DNA breaks had cohesive ends (Fig. 4B through E). Importantly, the amount of these cohesive end-bearing DNA breaks increased dramatically upon *Tb*TEL2 depletion (Fig. 4B and E). Specifically, significantly more DNA breaks have been detected at the active *VSG2* locus (Fig. 4B), which is prone to induce more VSG switching events (79, 80). Furthermore, LMPCR detected more DNA breaks at the silent *VSG21* (Fig. 4C), 70 bp repeats (Fig. 4D), and a chromosome-internal *SNAP50* locus (Fig. 4E) upon depletion of *Tb*TEL2. Furthermore, we prepared genomic DNA plugs from *TbTEL2*^F2H+/−^ RNAi cells and separated the undigested chromosome DNA by pulsed-field gel electrophoresis (PFGE). Individual megabase chromosomes are clearly visible after the PFGE gel was stained by ethidium bromide before and 24 h after inducing *Tb*TEL2 RNAi, but these DNA bands became much more smeared 48 and 72 h after the induction (Fig. S5E), indicating a significant amount of DNA degradation at later time points after depleting *Tb*TEL2. Therefore, *Tb*TEL2 is essential for genome integrity. We further analyzed the cell cycle profiles of *TbTEL2*^F2H+/−^ RNAi cells before and after inducing *Tb*TEL2 RNAi by FACS. Interestingly, *Tb*TEL2 depletion led to a significant increase in the percentage of G1-phase cells and significant decreases in the percentages of S, G2/M, and polyploid cells (Fig. S6).

### TbTEL2 maintains PIKK stability by suppressing the 26S proteasome-mediated degradation

Although *Tb*TEL2 depletion leads to more DNA breaks (Fig. 4B through E), we only detected a weak γH2A signal that disappeared quickly (Fig. 3G), suggesting that H2A kinases may not be fully active in *Tb*TEL2-depleted cells. Mouse TEL2 has been shown to maintain the protein levels of phosphatidylinositol-3 kinase-related kinases (PIKKs) (36). H2A kinases *Tb*ATR and *Tb*ATM (53) are both PIKK proteins, and *Tb*ATR plays a role in antigenic variation (55). Therefore, we examined whether *Tb*TEL2 is also critical to maintain normal protein levels of PIKKs.

**Fig 5:**
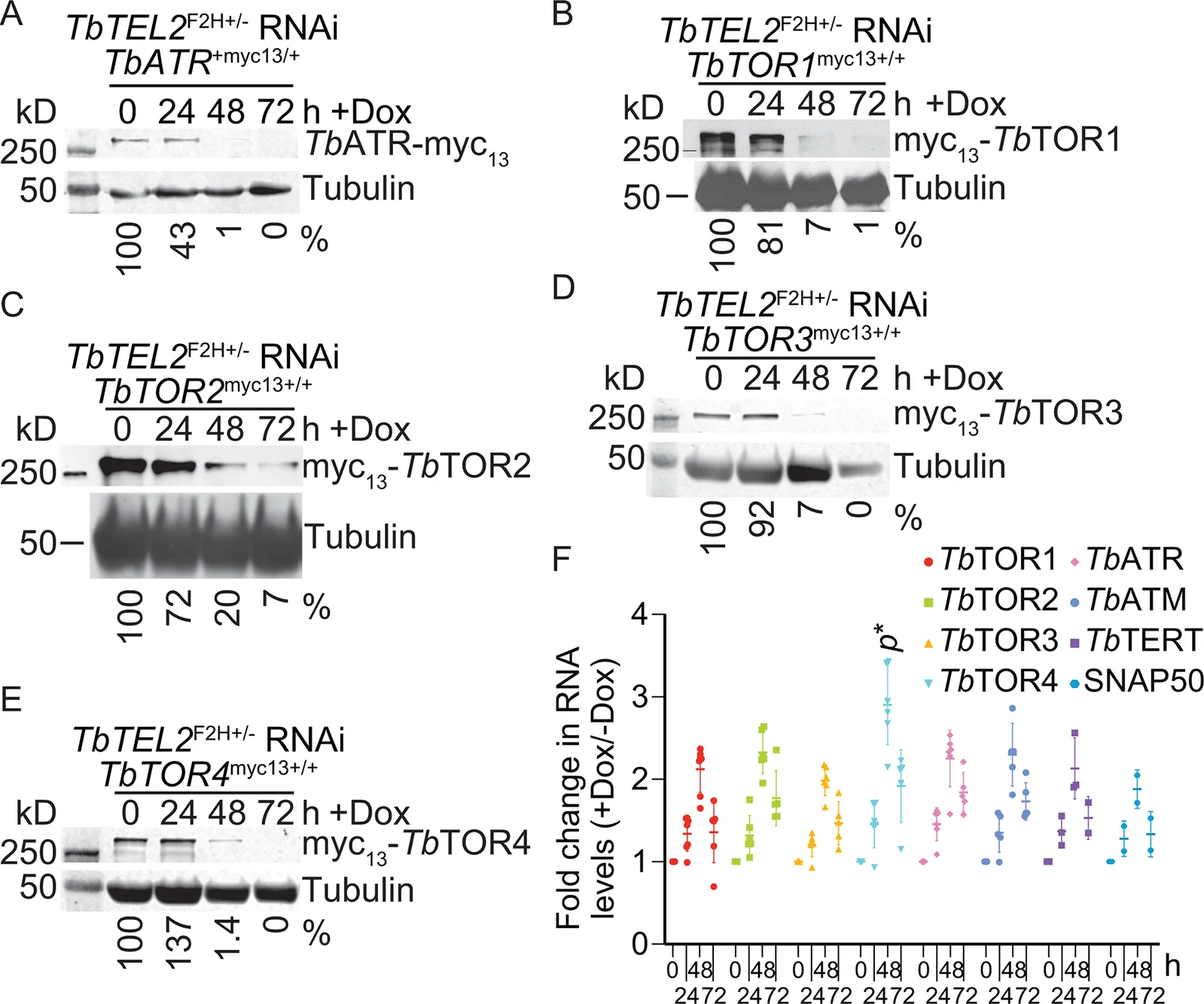
Western blotting analysis shows that depletion of *Tb*TEL2 leads to decreases in protein levels of *Tb*ATR (**A**), *Tb*TOR1 (**B**), *Tb*TOR2 (**C**), *Tb*TOR3 (**D**), and *Tb*TOR4 (**E**). Myc_13_-tagged proteins were detected using a mouse anti-myc antibody (9E10). Tubulin was detected as a loading control. Numbers at the bottom of each panel indicate relative protein levels (after normalized against the tubulin level). (**F**) Quantitative RT-PCR analysis shows that PIKK RNA levels did not decrease upon depletion of *Tb*TEL2. *, unpaired *t*-test *P* < 0.05 when RNA level changes are compared to that of the *TbTERT* RNA. Mean value and standard deviation for each time point are shown as horizontal and vertical bars.

*T. brucei* has six PIKK proteins, *Tb*ATR (*Tb*927.11.14680), *Tb*TOR1 (*Tb*927.10.8420), *Tb*TOR2 (*Tb*927.4.420), *Tb*TOR3 (*Tb*927.4.800), *Tb*TOR4 (*Tb*927.1.1930), and *Tb*ATM (*Tb*927.2.2260). Since antibodies recognizing these proteins are not available, we inserted a tag to one of the endogenous alleles of each PIKK except *Tb*ATM, whose multiple tagging attempts were not successful. In the *TbTEL2*^F2H+/−^ RNAi background, *Tb*ATR is C-terminally tagged with myc_13_, while *Tb*TOR1, *Tb*TOR2, *Tb*TOR3, and *Tb*TOR4 are N-terminally tagged with myc_13_ (Fig. S7). In these cells, we examined the PIKK protein levels before and after *Tb*TEL2 depletion. Western blotting using a mouse anti-myc antibody showed that the levels of these PIKK proteins decreased dramatically upon *Tb*TEL2 depletion (Fig. 5A through E). Therefore, *Tb*TEL2 helps maintain normal levels of PIKK proteins. However, qRT-PCR analysis showed that RNA levels of these PIKKs were not decreased upon depletion of *Tb*TEL2 (Fig. 5F and Table S4), suggesting that *Tb*TEL2 regulates PIKK protein levels at a post-transcriptional level.

**Fig 6:**
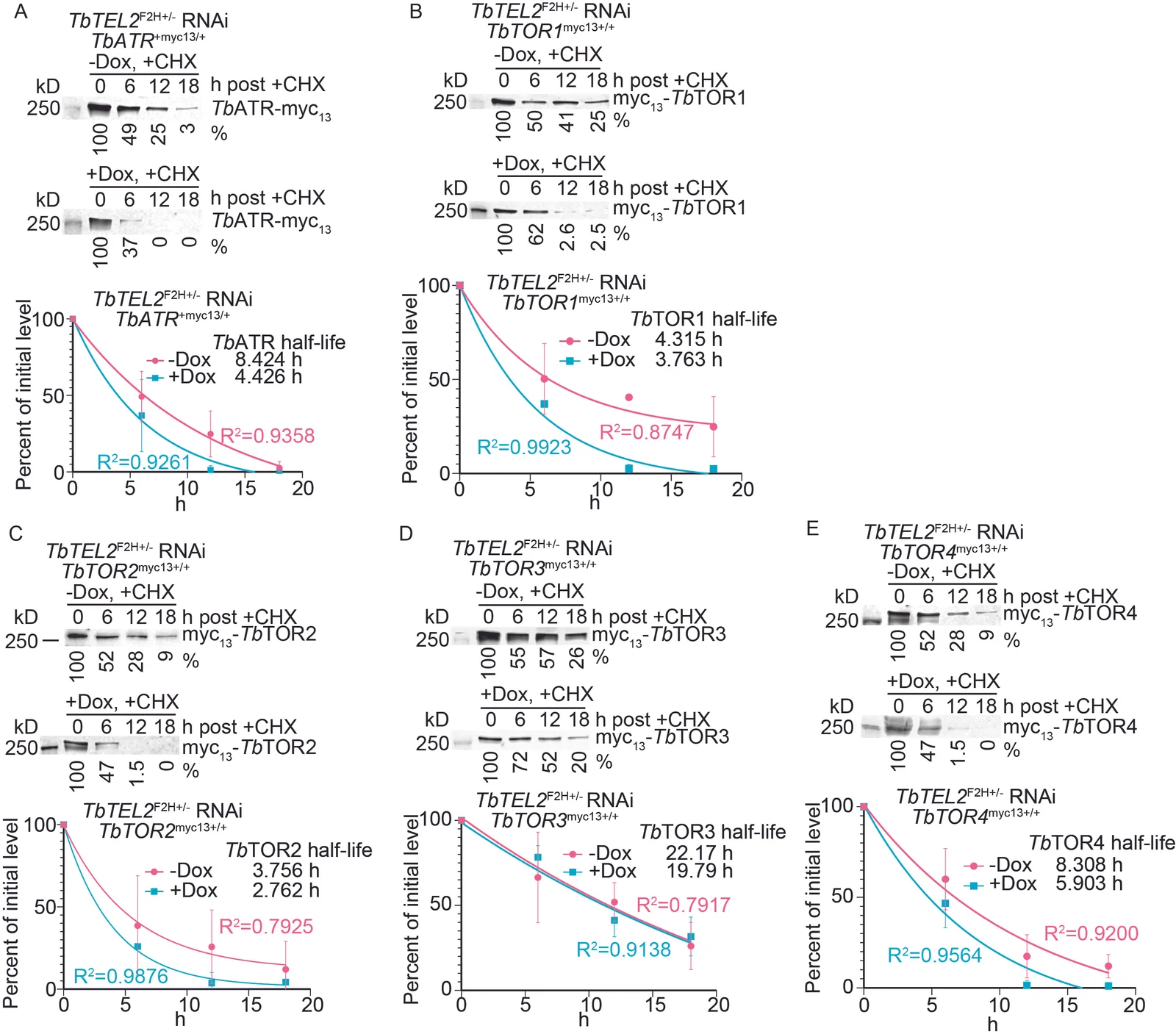
*Tb*TEL2 helps maintain protein stability of most PIKK proteins. Western blotting was performed to estimate protein levels of *Tb*ATR (**A**), *Tb*TOR1 (**B**), *Tb*TOR2 (**C**), *Tb*TOR3 (**D**), and *Tb*TOR4 (**E**) in uninduced and induced *TbTEL2*^F2H+/−^ RNAi cells that were treated with 100 µg/mL CHX for indicated length of time. Representative Western blotting results were shown (top of each panel). Protein decay curve was fit to the measured protein levels (quantified from three to five Western gels, Table S6) using GraphPad Prism and estimated half-lives are indicated (bottom of each panel). The *R*^2^ values for the goodness-of-fit analysis are indicated.

To explore the mechanisms of *Tb*TEL2’s function in PIKK protein level maintenance, we estimated the PIKK protein half-lives before and after *Tb*TEL2 depletion by inhibiting translation with cycloheximide (CHX) (25). A total of 100 µg/mL CHX was added to *TbTEL2*^F2H+/−^ RNAi cells before or 30 h after inducing *Tb*TEL2 RNAi. Whole-cell lysates were prepared at various time points after adding CHX and analyzed by Western blotting. Quantification of PIKK protein levels followed by protein decay analysis using GraphPad Prism showed that the half-lives of *Tb*ATR (Fig. 6A), *Tb*TOR1 (Fig. 6B), *Tb*TOR2 (Fig. 6C), and *Tb*TOR4 (Fig. 6E) are shorter in *Tb*TEL2-depleted cells than in uninduced *Tb*TEL2 RNAi cells (Fig. 6 and Table S6). In contrast, *Tb*TOR3’s half-life did not change significantly before and after depletion of *Tb*TEL2 (Fig. 6D and Table S6). Therefore, *Tb*TEL2 helps stabilize most PIKK proteins.

**Fig 7:**
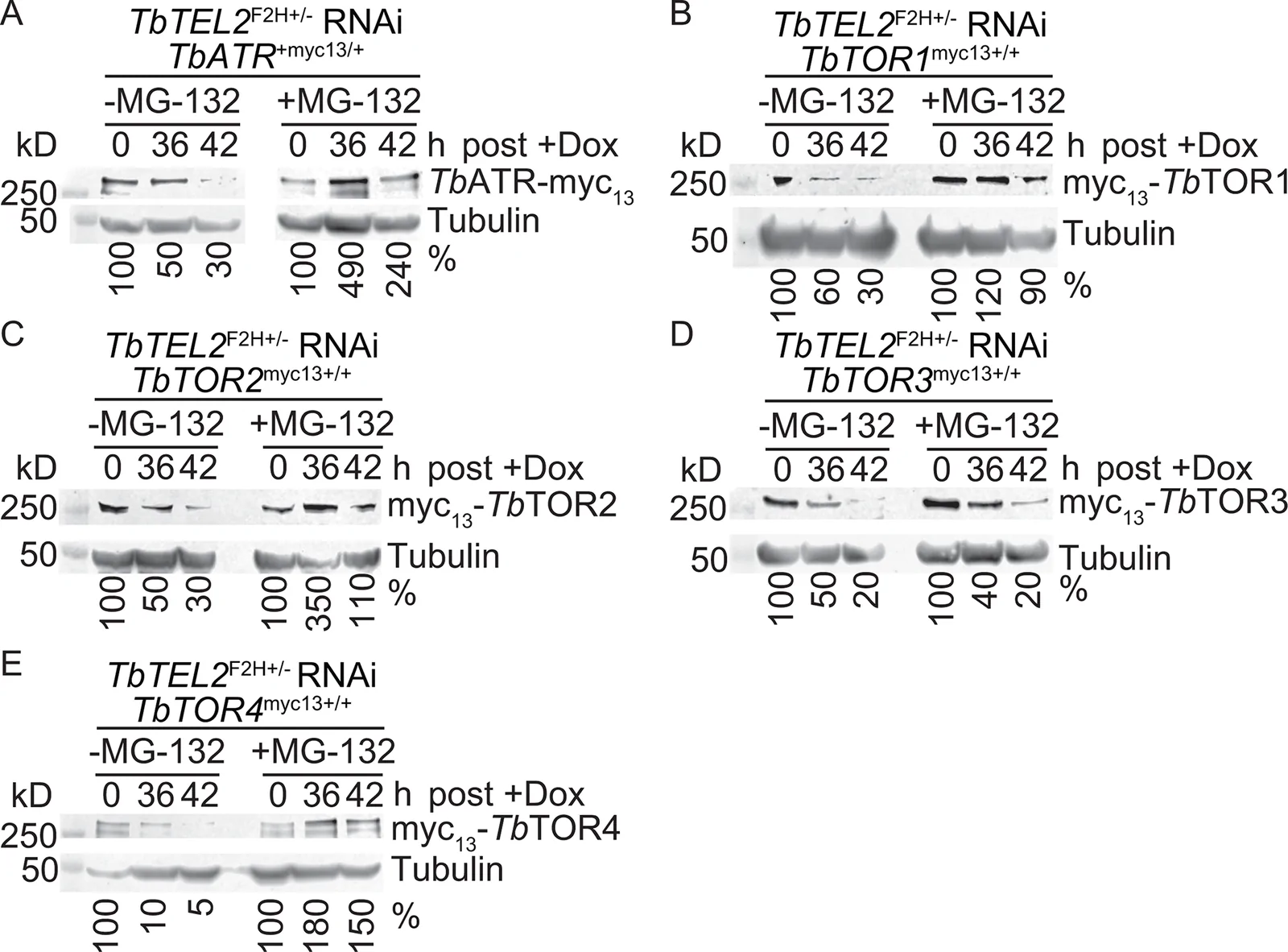
*Tb*TEL2 protects most PIKK proteins from the 26S proteasome-mediated degradation. Protein levels of *Tb*ATR (**A**), *Tb*TOR1 (**B**), *Tb*TOR2 (**C**), *Tb*TOR3 (**D**), and *Tb*TOR4 (**E**) were estimated by Western blotting in *Tb*TEL2-depleted cells treated with and without MG-132, which inhibits the 26S proteasome (25). Numbers at the bottom of each panel indicate relative protein levels (after normalizing against the tubulin level).

To investigate how *Tb*TEL2 helps maintain the PIKK protein stability, we further examined whether PIKK proteins were degraded by the 26S proteasome after *Tb*TEL2 was depleted. The PIKK protein levels were estimated by Western blotting before and after *Tb*TEL2 RNAi induction with and without treating cells with MG-132 (25), a 26S proteasome inhibitor, for 6 h. We found that after depleting *Tb*TEL2, *Tb*ATR (Fig. 7A), *Tb*TOR1 (Fig. 7B), *Tb*TOR2 (Fig. 7C), and *Tb*TOR4 (Fig. 7E) were degraded faster without the MG-132-mediated inhibition of the 26S proteasome than with MG-132 treatment. Therefore, *Tb*TEL2 suppresses the 26S proteasome-mediated degradation of *Tb*ATR, *Tb*TOR1, *Tb*TOR2, and *Tb*TOR4. On the other hand, the degradation of *Tb*TOR3 showed a similar rate in cells treated with and without MG-132 (Fig. 7D), indicating that *Tb*TEL2 regulates the *Tb*TOR3 protein level through a different mechanism.

Human TEL2 is constitutively phosphorylated at S487 and S491 residues by CK2, which is required for its direct interaction with PIH1D1, a subunit of the R2TP complex (52). In addition, the phosphorylation sites are conserved in vertebrate, worm, and yeast TEL2 homologs (Fig. S1E) (52). Furthermore, this interaction is critical for mammalian and yeast TEL2 homologs to act as a co-chaperone of HSP90 to ensure co-translational maturation of PIKKs (41, 48–52). However, kinetoplastid TEL2s do not have the conserved serine residues, even though the neighboring residues are clearly conserved with other TEL2 homologs (Fig. S1E). Therefore, although *T. brucei, T. cruzi,* and *Leishmania* clearly have a PIH1 homolog (*Tb*927.9.10490, *Tc*CLB.506147.150, and Ldbpk_354400.1, respectively) (81), it is unlikely that *Tb*TEL2, *Tc*TEL2, and *Lm*TEL2 interact directly with R2TP and act as co-chaperones of HSP90.

**Fig 8:**
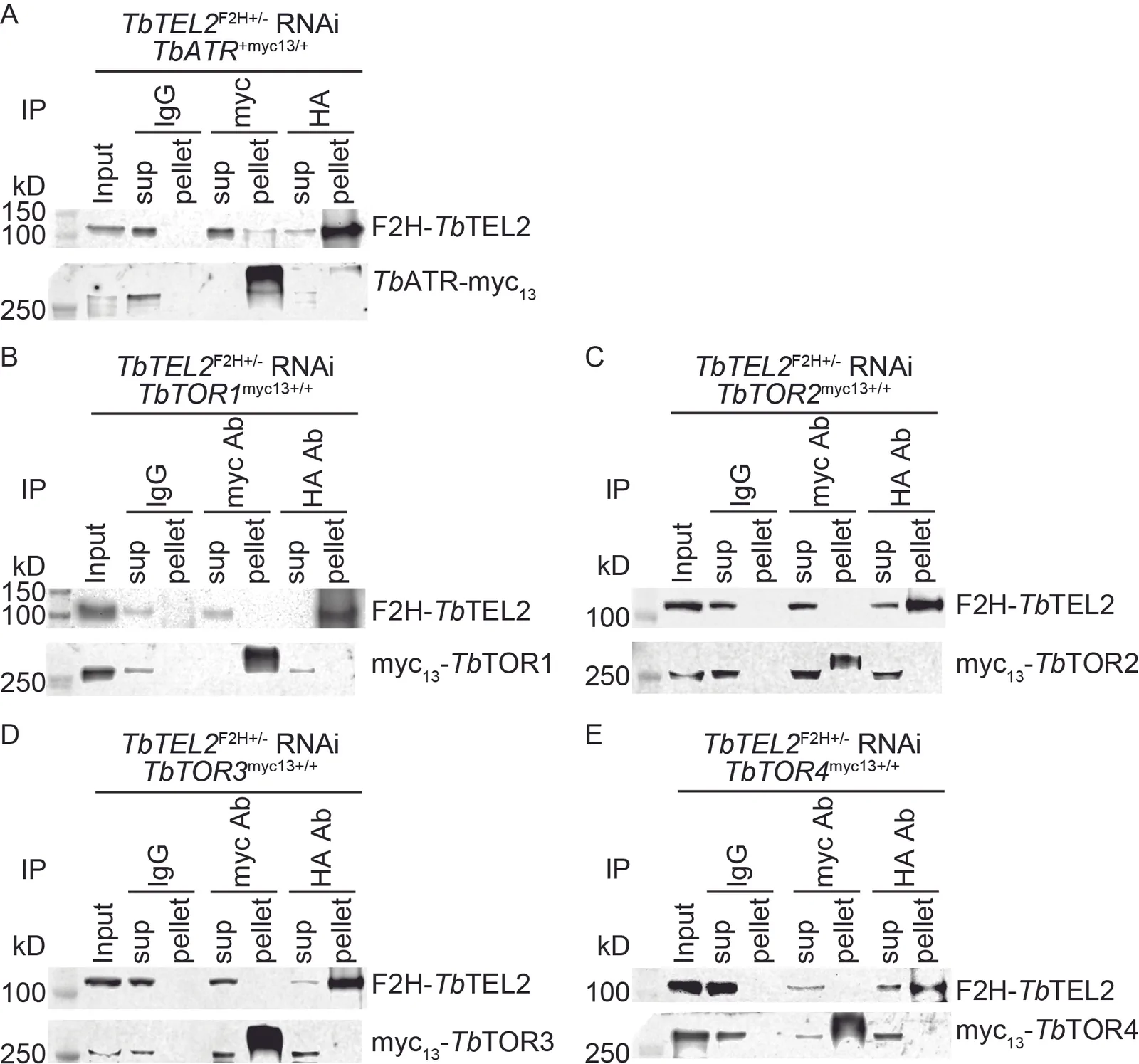
*Tb*TEL2 interacts with *Tb*ATR but not with *Tb*TOR1-4. IP experiments were performed in indicated strains using a mouse anti-myc antibody (9E10), a mouse anti-HA antibody (12CA5), and normal mouse IgG (as a negative control). Immunoprecipitated products were analyzed by Western blotting using a rabbit anti-myc antibody (GenScript) and a rabbit anti-HA antibody (Thermo Fisher Scientific). *Tb*TEL2 co-IPs with *Tb*ATR (**A**). Under the current IP condition, *Tb*TEL2 does not co-IP with *Tb*TOR1 (**B**), *Tb*TOR2 (**C**), *Tb*TOR3 (**D**), or *Tb*TOR4 (**E**).

TEL2 homologs have been shown to interact with many PIKK proteins in mammalian and yeast cells (36, 46, 51, 82), which is not affected by CK2-mediated phosphorylation (52). We therefore further investigated whether *Tb*TEL2 interacts with PIKK proteins in *T. brucei*. In *TbTEL2*^F2H+/−^ RNAi cells where PIKK proteins were tagged with myc_13_, we performed co-IP experiments to pull down the myc_13_-tagged PIKK proteins and F2H-*Tb*TEL2. We observed *Tb*TEL2 in the *Tb*ATR IP product and *Tb*ATR in the *Tb*TEL2 IP product (Fig. 8A). However, under the current IP conditions, we did not co-IP *Tb*TEL2 with *Tb*TOR1, *Tb*TOR2, *Tb*TOR3, or *Tb*TOR4 (Fig. 8B through E).

## DISCUSSION

We have identified *T. brucei* and *T. cruzi* TEL2 homologs, whose AlphaFold 3-predicted structures have two α-solenoids at the N- and C-terminal halves of the protein, which is typical of TEL2 homologs (51). Although sequence alignment of TEL2 homologs shows limited homology and identity, TEL2 homologs align reasonably well when their solved and predicted structures are compared, and the kinetoplastid TEL2 homologs have highly conserved structures. In addition, all predicted TEL2 homolog structures have pTM values much greater than 0.5, indicating high confidence in the predicted homology among these proteins.

We have shown that *Tb*TEL2 is essential for cell proliferation, as depleting *Tb*TEL2 leads to parasite growth arrest. However, several observations suggest that *Tb*TEL2 is not one of the major components of the *T. brucei* telomere protein complex. First, perturbation of the *Tb*TEL2 level by RNAi or expression of an ectopic *TbTEL2* allele does not affect telomere growth rates. Second, *Tb*TEL2 does not interact with *Tb*RAP1 or *Tb*TIF2, two key telomere proteins in *T. brucei* (6, 11, 26). Nor does *Tb*TEL2 affect the protein level of *Tb*TRF, a key component of the *T. brucei* telomere complex (24). Third, only two *Tb*TEL2 peptides were identified in the telomere PICh experiment with a low Sequest score (30). Fourth, it is worth noting that although *Sc*TEL2 was originally identified due to the short telomere phenotype in the *tel2-1* mutant (33), none of the known TEL2 homologs have been shown to be tightly associated with the telomere chromatin. Therefore, *Tb*TEL2 is likely not a core component of the telomere protein complex, although we cannot exclude the possibility that *Tb*TEL2 may be associated with the telomere chromatin transiently under certain conditions.

We found that depletion of *Tb*TEL2 disrupts the strict VSG monoallelic expression and increases VSG switching rate, which is linked to *Tb*TEL2’s role in maintaining genome integrity, as we detected more DNA breaks at both telomeric and chromosome-internal loci by LMPCR. Although depletion of *Tb*TEL2 leads to an elevated TERRA level (~2-fold), this is unlikely the key reason for more DNA breaks in the genome. First, compared to the consequences of depletion of *Tb*RAP1 or *Tb*TRF, which lead to ~10-fold increase in the TERRA level (8, 28), the effect of *Tb*TEL2 depletion on TERRA is much milder and may not induce a dramatic increase in the TRL level. Second, we detect more DNA breaks at multiple loci, including a chromosome-internal locus, which is unlikely induced by the TRL structure. Nevertheless, in *Tb*TEL2-depleted cells, we detected more DNA breaks at the active *VSG* vicinity, which has been shown to be a potent trigger for VSG switching (79, 80). Interestingly, depletion of *Tb*TEL2 only increases the γH2A level transiently and mildly. Similarly, *Tb*RAD51 protein level was increased and peaked at 48 h after inducing *Tb*TEL2 RNAi. Yet, in the absence of a persistent DNA damage signaling through γH2A, loss of genome integrity still induces a robust increase in VSG switching rate. Therefore, our observations strongly suggest that the physical DNA breaks are a key to trigger more VSG switching events. In addition, alternative DNA damage response mechanisms independent of γH2A signaling may be activated upon *Tb*TEL2 depletion, which subsequently leads to more VSG switching (see below).

We found that *Tb*TEL2, like other TEL2 homologs, also plays an important role in maintaining the stability of PIKK proteins, indicating that this is an essential conserved function of TEL2 homologs. PIKK proteins regulate multiple cellular processes, including cell growth, cell survival, and cell response to environmental stimuli (43). Specifically, depletion of *Tb*TOR1 leads to accumulation of G1 phase cells (56). We also observed that *Tb*TEL2 depletion leads to the same phenotype, presumably due to the decreased *Tb*TOR1 protein level in *Tb*TEL2-depleted cells. Our and others’ findings make TEL2 homologs central regulators essential for cell viability. Interestingly, we found that inhibiting the 26S proteasome activity by MG-132 can better stabilize protein levels of *Tb*ATR, *Tb*TOR1, *Tb*TOR2, and *Tb*TOR4 after *Tb*TEL2 is depleted, indicating that *Tb*TEL2 protects these proteins from the 26S proteasome-mediated degradation. This is different from what has been observed in mammalian and yeast cells. Specifically, mammalian TEL2 does not inhibit proteasome-mediated PIKK degradation (36). Instead, mammalian and fission yeast TEL2 homologs interact with TTI1 and TTI2 to form a heterotrimeric complex TTT (83), and TTT acts as a co-chaperone of HSP90 via its interaction with the R2TP complex to ensure co-translational maturation of PIKK proteins (51, 52, 84). Using human and budding yeast TTI1 and TTI2 as queries, we performed BLASTP searches in the *T. brucei* genome but did not identify any TTI homologs. However, *T. brucei* is a very early-branching eukaryote, and its protein complexes with conserved functions can have kinetoplastid-specific subunits. For example, the *Tb*ORC complex that is essential for DNA replication initiation has only two subunits (ORC1 and ORC4) with discernible sequence homology with their counterparts in mammals and yeasts (85, 86). The other four *Tb*ORC subunits are parasite-specific (86). It is possible that *Tb*TEL2 may interact with proteins with TTI1/2-like functions, but they have little sequence homology to known TTI proteins. *T. brucei* has the R2TP complex (81, 87). However, we found that *Tb*TEL2 lacks the conserved serine residues identified in vertebrate and yeast TEL2 homologs, whose phosphorylation by CK2 is required for their interaction with PIH1 homologs. Therefore, although we cannot completely exclude the possibility that *Tb*TEL2 may act as an HSP90 co-chaperone, it is unlikely that *Tb*TEL2 interacts with PIH1 directly. Considering all our observations, we conclude that *Tb*TEL2 helps maintain the stability of most PIKK proteins by suppressing the 26S proteasome-mediated degradation. It is also apparent that *Tb*TEL2 uses different mechanism(s) to help maintain the *Tb*TOR3 protein level. Nevertheless, it is unlikely that *Tb*TEL2 interacts with R2TP directly and acts as an HSP90 co-chaperone in the same way as TEL2 homologs in vertebrates and yeasts.

As described above, depletion of *Tb*TEL2 results in an increased amount of DNA damage in the genome but only increases the γH2A level transiently. It has been shown that phosphorylation of H2A (into γH2A) in response to ionizing irradiation is mostly mediated by *Tb*ATM and partly mediated by *Tb*ATR (53). Here, the *Tb*TEL2-depleted cells were not treated with ionizing irradiation, and DNA breaks can result from intrinsic factors such as DNA replication stress and reactive oxygen species. It is possible that sensing these types of DNA damage relies more on *Tb*ATR, and the loss of *Tb*ATR in *Tb*TEL2-depleted cells blocks the generation of γH2A even in the presence of DNA breaks. In addition, depletion of *Tb*ATR has been shown to alter RNAP I-transcribed *VSG* gene expression and increase genome instability (55). *Tb*TEL2 depletion clearly diminishes the *Tb*ATR protein level, which can contribute to the loss of VSG monoallelic expression and elevated VSG switching rate phenotypes. Although we could not examine whether *Tb*TEL2 also helps maintain *Tb*ATM’s protein level, *Tb*ATM has not been reported to play a role in antigenic variation. In addition, *T. brucei* has a KKIP5-dependent, ATM/ATR-independent DNA damage response pathway (54). This pathway may be activated upon *Tb*TEL2 depletion and thereby increase the VSG switching rate.

Under the current condition, we found that *Tb*TEL2 interacts with *Tb*ATR but not with *Tb*TOR1-4, suggesting that *Tb*TEL2 behaves more similarly to *Sc*TEL2 that interacts with Tel1 (ATM homolog) and Mec1 (ATR homolog). In addition, although the *Tb*TOR3 protein level decreases in *Tb*TEL2-depleted cells, *Tb*TOR3’s half-life is not significantly affected by *Tb*TEL2 depletion, and inhibiting the 26S proteasome activity does not rescue the *Tb*TOR3 protein level in *Tb*TEL2-depleted cells. Therefore, even within *T. brucei*, *Tb*TEL2 appears to regulate different PIKK protein levels using different mechanisms.

In summary, we have identified the *T. brucei* TEL2 homolog that is predicted to fold into a structure highly conserved with those of known TEL2 homologs. We have shown that *Tb*TEL2 has the same function as other TEL2 homologs in maintaining the stability of most PIKK proteins and that *Tb*TEL2 helps maintain genome stability and suppresses VSG switching, indicating that *Tb*TEL2 is a master regulator for parasite survival. However, unlike TEL2 homologs in vertebrates and yeasts, *Tb*TEL2 protects four PIKKs from the 26S proteasome-mediated protein degradation. Our study reveals conserved and unique mechanisms of *Tb*TEL2’s functions, indicating that *Tb*TEL2 can serve as a promising target for anti-parasite agents.

## MATERIALS and METHODS

### *T. brucei* strains and plasmids

All *T. brucei* strains used in this study are derived from bloodstream-form Lister 427 cells (single marker, also known as SM), which express T7 polymerase and the Tet repressor (88). All strains express VSG2. All *T. brucei* cells were cultured in the HMI-9 medium supplemented with 10% FBS and appropriate antibiotics. Table S3 lists all *T. brucei* strains used in this study. The parent strain, transfected plasmid, and selectable marker are also listed in Table S3.

### Quantitative real-time PCR (qRT-PCR)

Quantitative RT-PCR was performed as described previously (6, 9, 11). Total RNA was isolated from *T. brucei* cells using RNAstat-60 (TelTest, Inc.), treated by DNase (Qiagen), and purified using the RNeasy kit (Qiagen). For all RNA samples, OD_260_/OD_280_ values were greater than 1.8. cDNA was synthesized using a random hexamer and the MMLV reverse transcriptase (Promega) according to the manufacturer’s manual. In each subsequent qPCR reaction, 2 µL of cDNA was used after the cDNA product was diluted 1:100 (for *TERT*, tubulin, 18S rRNA, and *VSG2*), 1:10 (for *Tb*ATR, *Tb*TOR1, *Tb*TOR2, *Tb*TOR3, *Tb*TOR4, and *SNAP50*), or 1:1 (for all silent *VSG* genes). cDNAs were analyzed by real-time qPCR on a CFX Connect (Bio-Rad) using SsoAdvanced Universal SYBR Green Supermix (Bio-Rad) according to the manufacturer’s manual. rRNA and tubulin levels were measured and used as a loading control. Data acquired on CFX Connect were processed using MS Excel and GraphPad Prism. qPCR primer sequences are listed in Table S7.

### VSG switching assay

The VSG switching assay was performed as described in reference 26 with minor changes. Specifically, S/ev and S/*Tb*TEL2-SKOi cells (induced for 48 h) were cultured for ~10.5 population doublings. At the end of culturing, 30 million cells were incubated with 10 µg of VSG2 monoclonal antibody (23) on ice for 15 min. After washing three times with growth medium, cells were incubated with MACS beads conjugated with a rat anti-mouse antibody (Miltenyi) on ice for 15 min, followed by washing with growth medium twice. The mixture was then loaded onto an LD column, and cells in the flow-through fraction were collected and plated on 96-well dishes. One-sixth of the collected cells (equivalent to 5 million cells of the initial population) were evenly distributed onto three 96-well dishes. Similarly, 1/3 (equivalent to 10 million cells of the initial population) and 1/2 (equivalent to 15 million cells of the initial population) of the collected cells were evenly distributed into six and eight 96-well dishes, respectively. All recovered clones were tested again by Western slot blot using a VSG2-specific rabbit antibody, and VSG2-positive clones were excluded from true switchers. Raw switching frequency was calculated by dividing the number of true switchers by the initial cell number. To determine plating efficiency, cells were plated at 1 cell/well concentration onto 3 × 96-well plates. Plating efficiency was calculated by dividing the number of clones grown up by 288. Final switching rate was calculated by normalizing raw switching frequency with plating efficiency and dividing by the number of population doublings. Data were processed using MS Excel and GraphPad Prism.

### Telomere southern blotting and in-gel hybridization

To determine the bulk telomere length, genomic DNA was isolated from *T. brucei* cells by phenol-chloroform extraction and isopropanol precipitation without vortexing to preserve long DNA fragments (24, 89–91). Genomic DNA was digested with AluI and MboI, and 1 µg of digested DNA/lane was separated by agarose gel electrophoresis until the 1.5 kb DNA marker band reaches the bottom of a 20 cm gel. For telomere Southern blotting, the gel was sequentially washed in 0.25 M HCl, denaturing buffer (1.5 M NaCl/0.5 M NaOH), neutralizing solution (3 M NaCl/0.5 M Tris•Cl, pH 7.0), and 20× SSC. The DNA was subsequently blotted onto a Hybond nylon membrane, followed by UV cross-linking. The membrane was then hybridized with a radioactive 800 bp (TTAGGG)_n_ probe. For in-gel hybridization, the agarose gel containing gDNA was dried by vacuuming at room temperature overnight. The dried gel was then washed in denaturing buffer, neutralizing solution, and 20× SSC, followed by hybridization with a radioactive (CCCTAA)_4_ oligo probe. After washing away non-specific hybridization signals, the membrane/gel was exposed to a phosphorimager.

### Co-immunoprecipitation

Three hundred million log-phase growing cells were harvested by centrifugation and resuspended in lysis buffer (50 mM Tris•Cl pH 7.5, 0.5% NP-40, 2 mM EDTA, 20% glycerol, 0.15 M NaCl, 1× protease inhibitor [Sigma], 1 mM PMSF, 1 mM DTT, 4 µg/mL pepstatin A, 0.5 mg/mL TLCK). Glass beads were added to the cell suspension, and cells were sheared by vortexing. Clear whole-cell lysate was incubated with appropriate antibody or normal IgG (as a negative control). IP products were pulled down by Dynabeads Protein G (Thermo Fisher Scientific). One percent of input sample was loaded as a control. IP products were subsequently analyzed by Western blotting.

### MG-132 treatment

*T. brucei* cells were incubated in medium with 25 μM of MG-132 for 6 h before proteins were extracted for Western analysis according to (25, 92). For RNAi induced samples, MG-132 was also added to the medium 6 h prior to the time point of harvesting cells.

### Cycloheximide (CHX) chase assay

*Tb*TEL2 RNAi cells with appropriate myc_13_-tagged PIKK protein were either induced by 100 ng/mL doxycycline for 30 h or uninduced when 100 µg/mL CHX was added according to references 93–95. Whole-cell lysate was prepared at 0, 6, 12, and 18 h after adding CHX, followed by Western blotting analysis.

### Ligation-mediated PCR

LMPCR was performed as described in references 96, 97. Briefly, in each ligation reaction, 2 μg of genomic DNA was either treated or not treated with 2 μL of T4 DNA polymerase (3,000 U/mL, New England BioLabs) in the presence of 200 μM dNTP and then ligated with 10 μL annealed adaptor. The LMPCR adaptor was prepared by annealing 5 nmole of LMPCR-Linker-long (5′-GCGGTGACCCGGGAGATCTGAATTCAC-3′) and 5 nmole of LMPCR-Linker-short (5′-GTGAATTCAGATC-3′) primers in 150 µL of 1× ligase buffer. Three 1:3 serial dilutions of the ligated products were prepared and used in subsequent PCR using Hotstart Platinum Taq DNA polymerase (Thermo Fisher Scientific) and a touchdown PCR program. PCR products were separated by electrophoresis, and Southern in-gel hybridization was performed using locus-specific radioactive oligonucleotide probes. The serial diluted genomic DNAs were also used as a template for PCR reactions using the *Tb*RAP1 primers, and the PCR products were run as a loading control. One set of loading control was prepared for the same genomic DNA sample used for Southern in-gel hybridizations using different locus-specific probes. Primers used in LMPCR are listed in Table S8.

### FACS analysis

*TbTEL2*^F2H+/−^ RNAi cells induced for 0, 24, 48, and 72 h were fixed in ice-cold EtOH. Right before FACS analysis, cells were stained with propidium iodide. FACS was performed on a BD FACSymphony A5 at the Flow Cytometry Core of Cleveland Clinic Research.

### Pulse-field gel electrophoresis (PFGE)

DNA plugs were prepared from *TbTEL2*^F2H+/−^ RNAi cells induced for 0, 24, 48, and 72 h at 250 million cells/mL in low-melting-point agarose. PFGE was performed in a 0.5 × TBE/1.2% agarose gel using a Bio-Rad CHEF DR II or a CHEF DR III unit with the following conditions: initial pulse 1500 s, ending pulse 700 s, voltage 2.5 V/cm, running time 144 h, and temperature 14°C.

### Statistical analyses

Unpaired Student *t*-tests were performed using GraphPad Prism to compare different phenotypes observed in induced (+Dox) and uninduced (−Dox) samples in Fig. 3A, D, F and 5F; Fig. S6B. Goodness-of-fit analysis was performed in GraphPad Prism when protein decay curves were fitted to the measured PIKK protein levels to generate Fig. 6. To examine the relationship between telomere growth rate vs telomere fragment size in WT cells, we performed the following tests: Pearson correlation (linear inverse association X–Y), Spearman rank correlation (monotone inverse association), simple linear regression *Y* on *X,* and regression using 1/*X* to test an inverse-proportional form. Analyses were performed in Python using the following libraries: pandas for data handling and SciPy (scipy.stats) for Pearson/Spearman correlations and linear regressions (both with X and with 1/*X* as predictors). To compare two groups of telomere growth data from induced (+Dox) and uninduced (−Dox) GFP-*Tb*TEL2 cells, we modeled the relationship between telomere fragment size (TFS) and telomere growth rate (TGR) using linear regression with the induction condition (IC, either 0 or 1 for the two conditions) and an interaction term (TFS x IC) within an ANCOVA framework:

TGR = *β_0_ + β_1_*TFS + *β_2_*IC + *β_3_*(TFS × IC)

Models were fit in Python (statsmodels) and evaluated for an interaction effect (difference in slopes) and a main effect of IC (intercept shift).

To compare the distributions of VSG switching mechanisms in S/*Tb*TEL2-SKOi cells and S/ev cells, all switchers and their determined switching mechanisms (Table S5) were pooled to generate a 2 × 5 contingency table with five categories (ES GC/ES loss + *in* situ, VSG GC, crossover, *in situ*, and other). An exact Monte Carlo test (Fisher–Freeman–Halton type) was performed using a Python script with NumPy to simulate contingency tables under the null and to compute a chi-square-type statistic. The null hypothesis is that the VSG switching mechanism distributions are the same between the two cell types, and rejection implies that the patterns differ.

## Supplementary Material

Reviewer comments

## SUPPLEMENTAL MATERIAL

Supplemental material spectrum.03516-25-s0001.pdf Fig. S1 to S7; Tables S1 to S3, S7, and S8.

Table S4 spectrum.03516-25-s0002.xlsx RT-PCR raw data.

Table S5 spectrum.03516-25-s0003.xlsx VSG switching assay details.

Table S6 spectrum.03516-25-s0004.xlsx Raw quantification data for Fig. 6.

OPEN PEER REVIEW reviewer-comments.pdf An accounting of the reviewer comments and feedback.

ASM does not own the copyrights to Supplemental Material that may be linked to, or accessed through, an article. The authors have granted ASM a non-exclusive, world-wide license to publish the Supplemental Material files. Please contact the corresponding author directly for reuse.
