# Supplementary material for: Trypanosoma brucei TEL2 inhibits VSG switching and protects PIKKs from the 26S proteasome-mediated degradation
Source: Microbiol Spectr. 2026 May 29;14(7):e03516-25. doi: 10.1128/spectrum.03516-25 (PMC13340017; doi:10.1128/spectrum.03516-25)
Supplement: Supplemental material — Fig. S1 to S7; Tables S1 to S3, S7, and S8. [file spectrum.03516-25-s0001.pdf]

Sayeed et al. Fig. S1

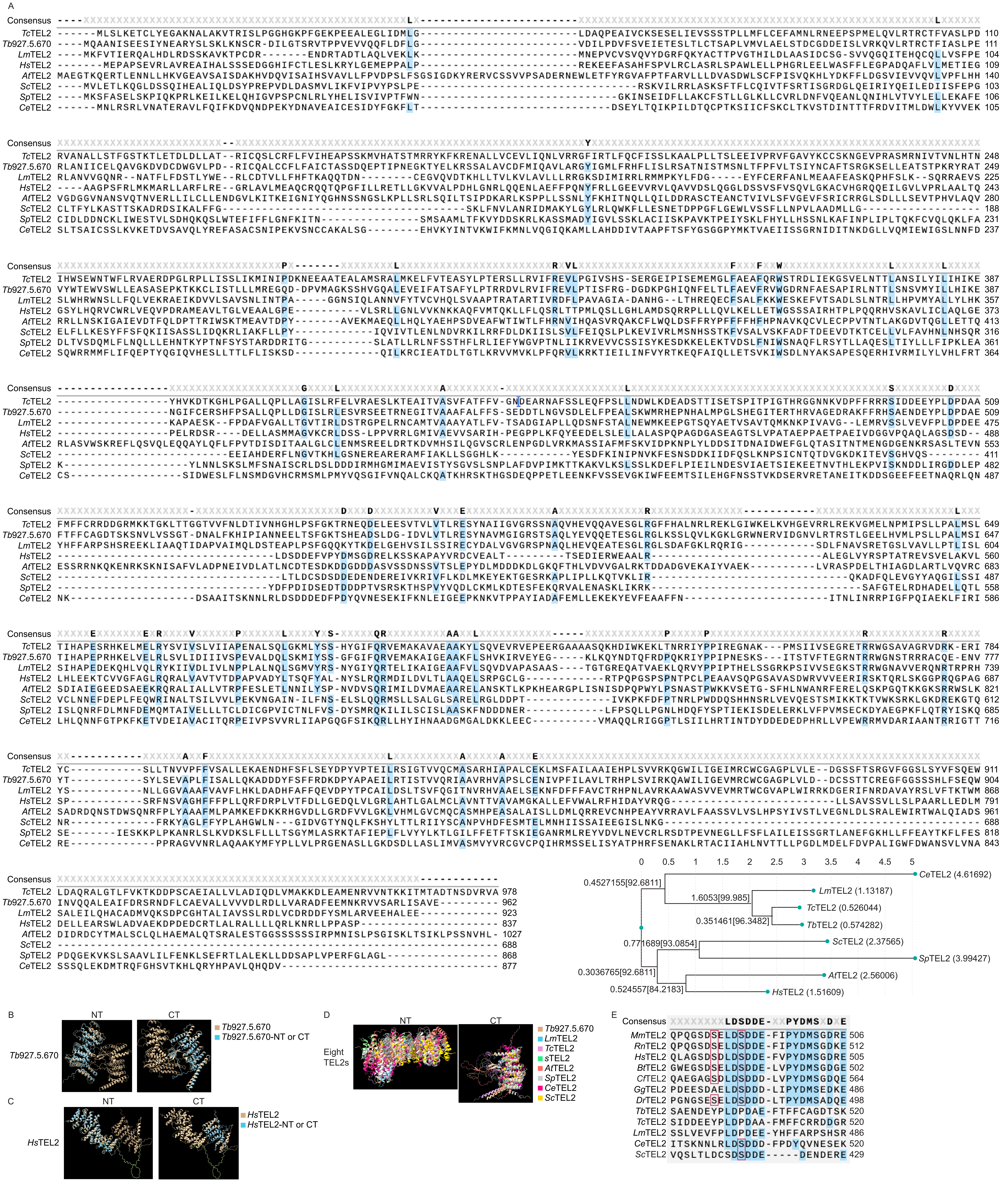

Sayeed et al. Fig. S2

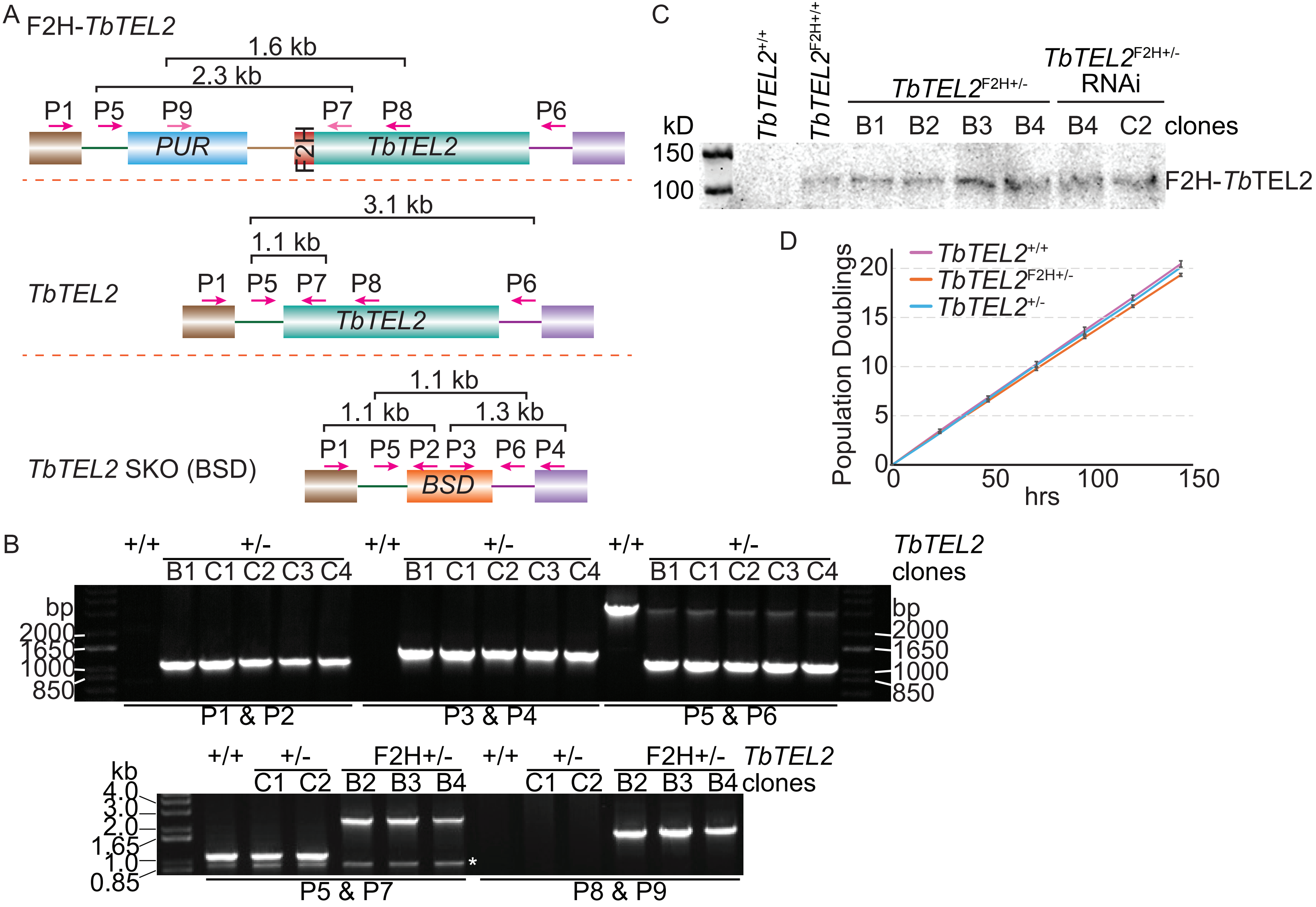

Sayeed, et al. Fig. S3

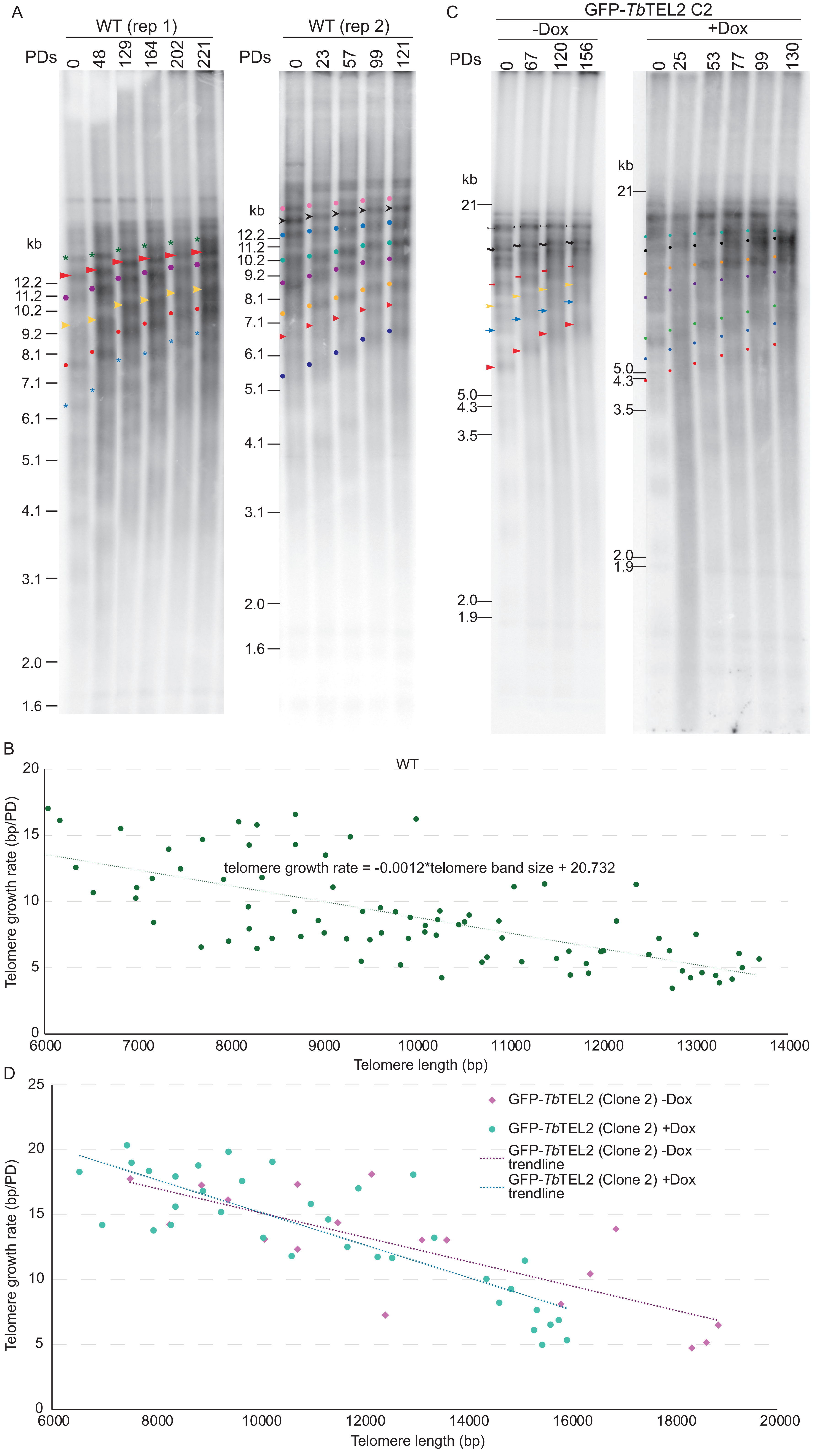

Sayeed et al. Fig. S4

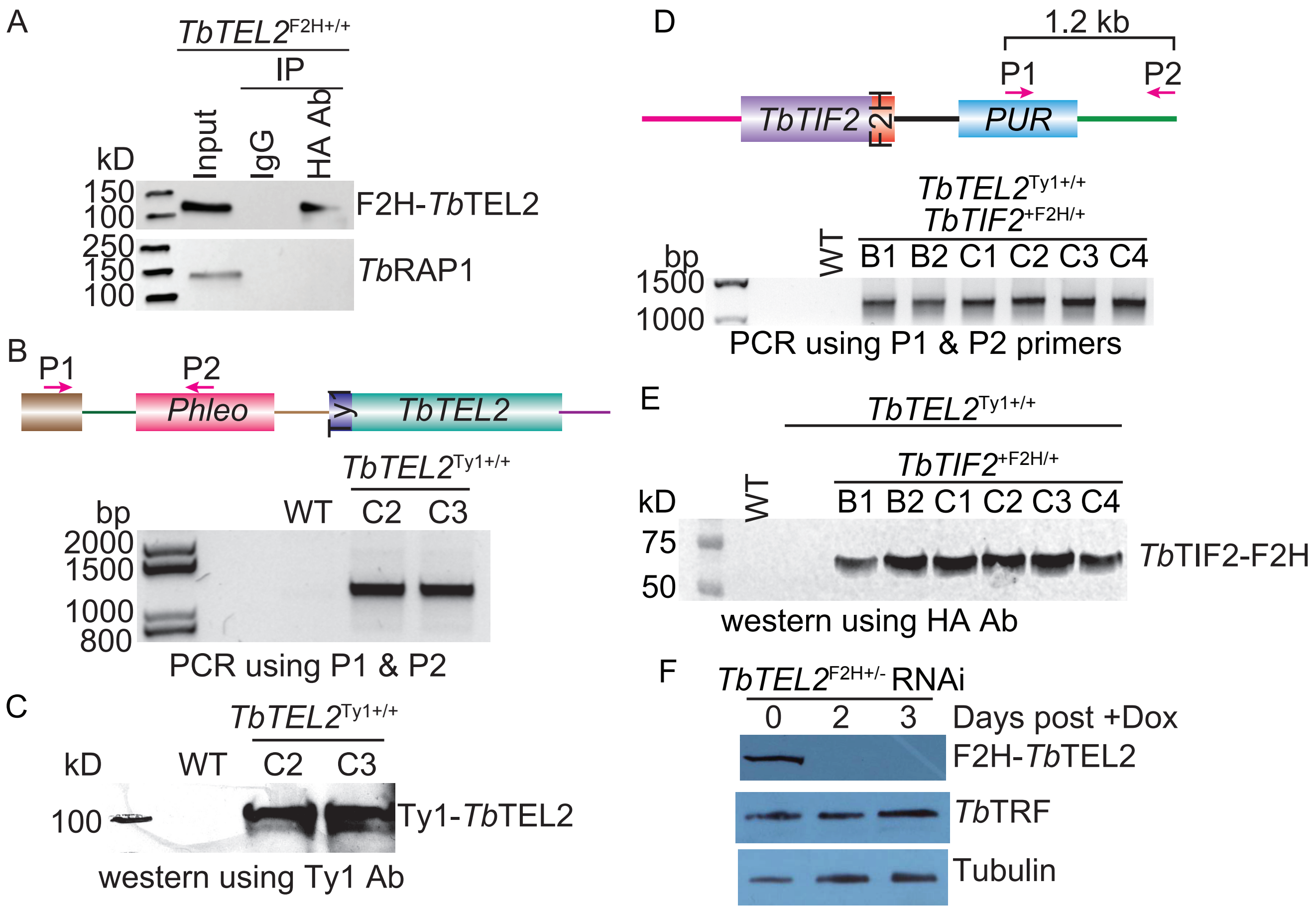

Sayeed et al. Fig. S5

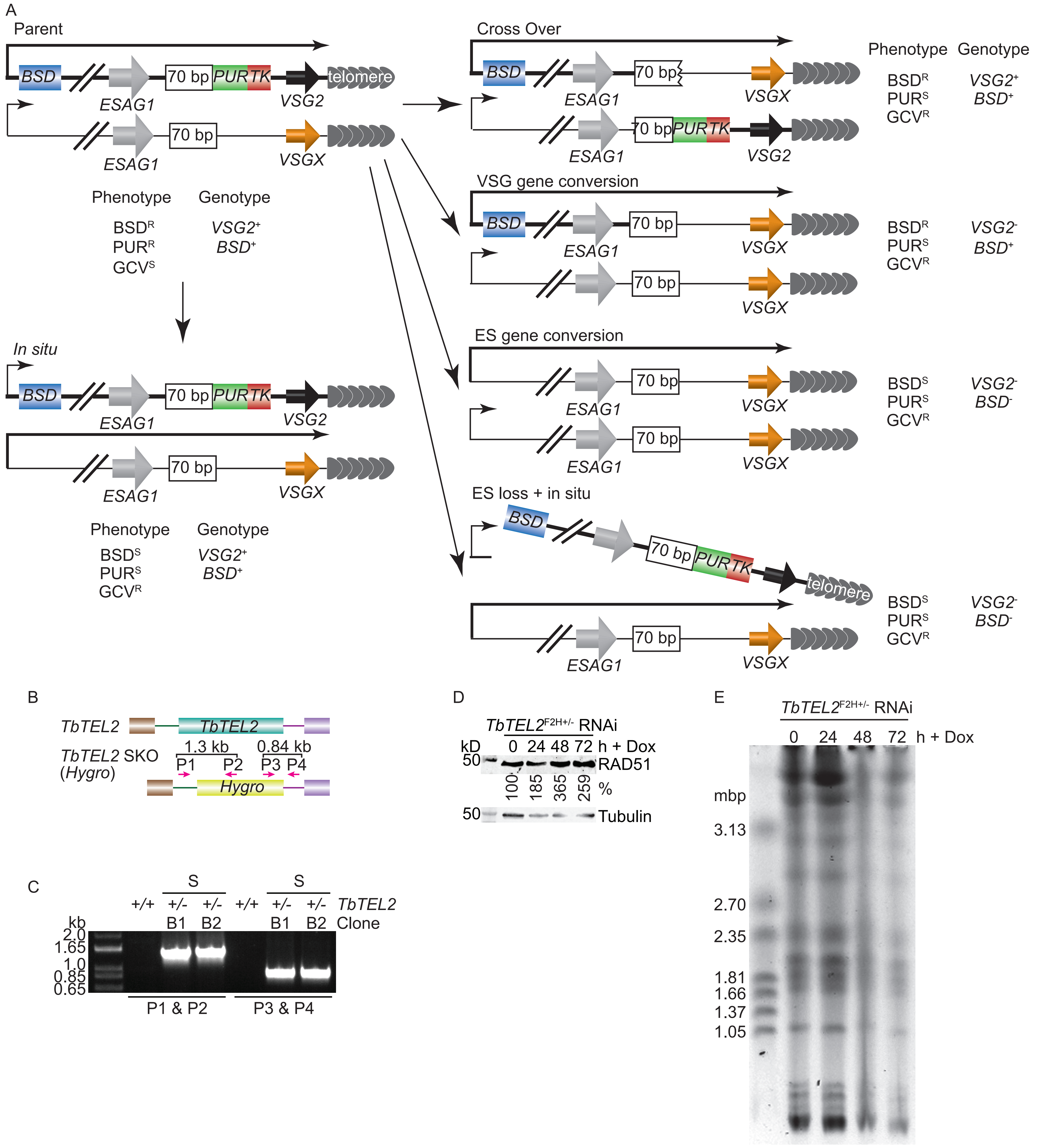

Sayeed et al. Fig. S6

A

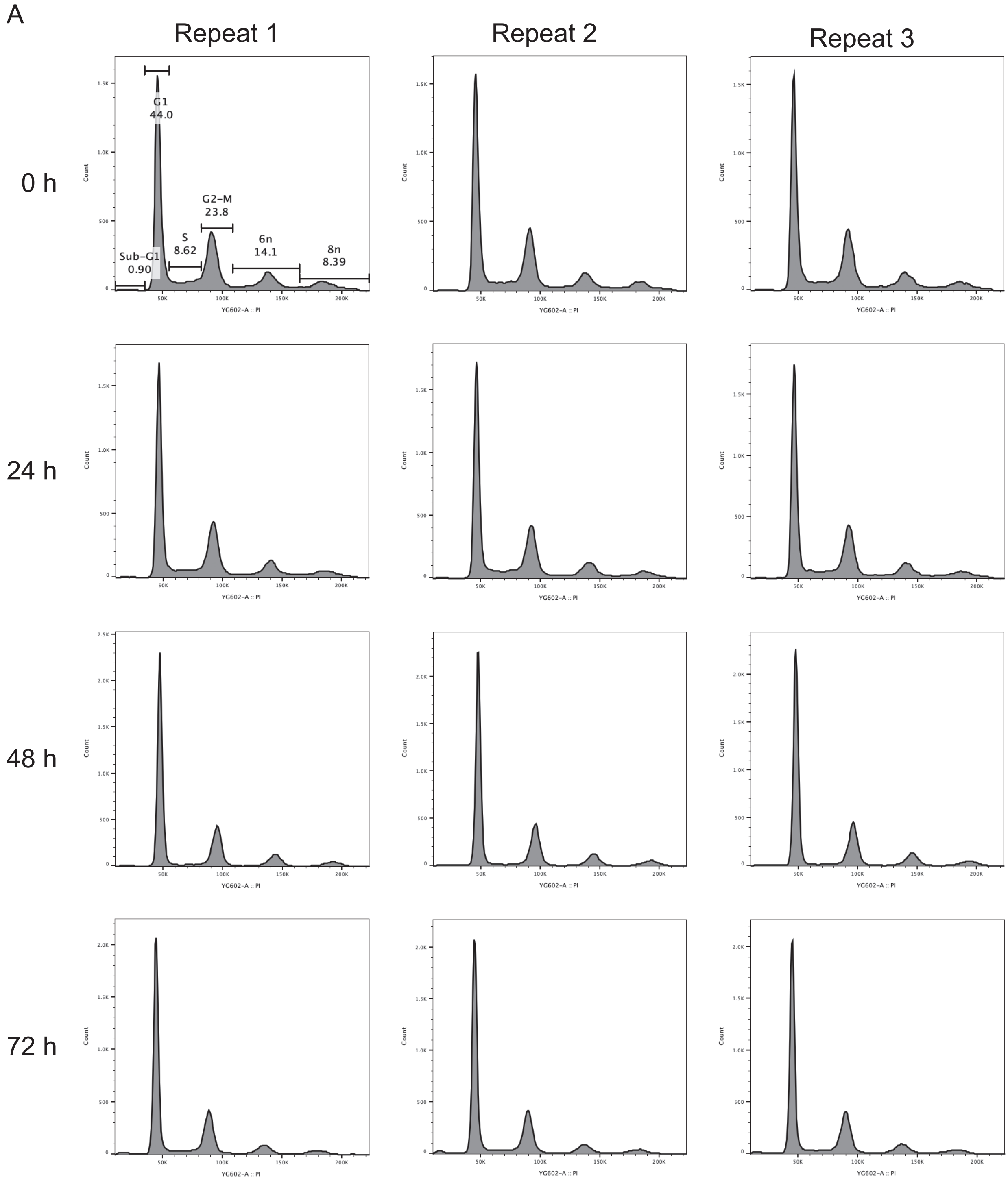

B

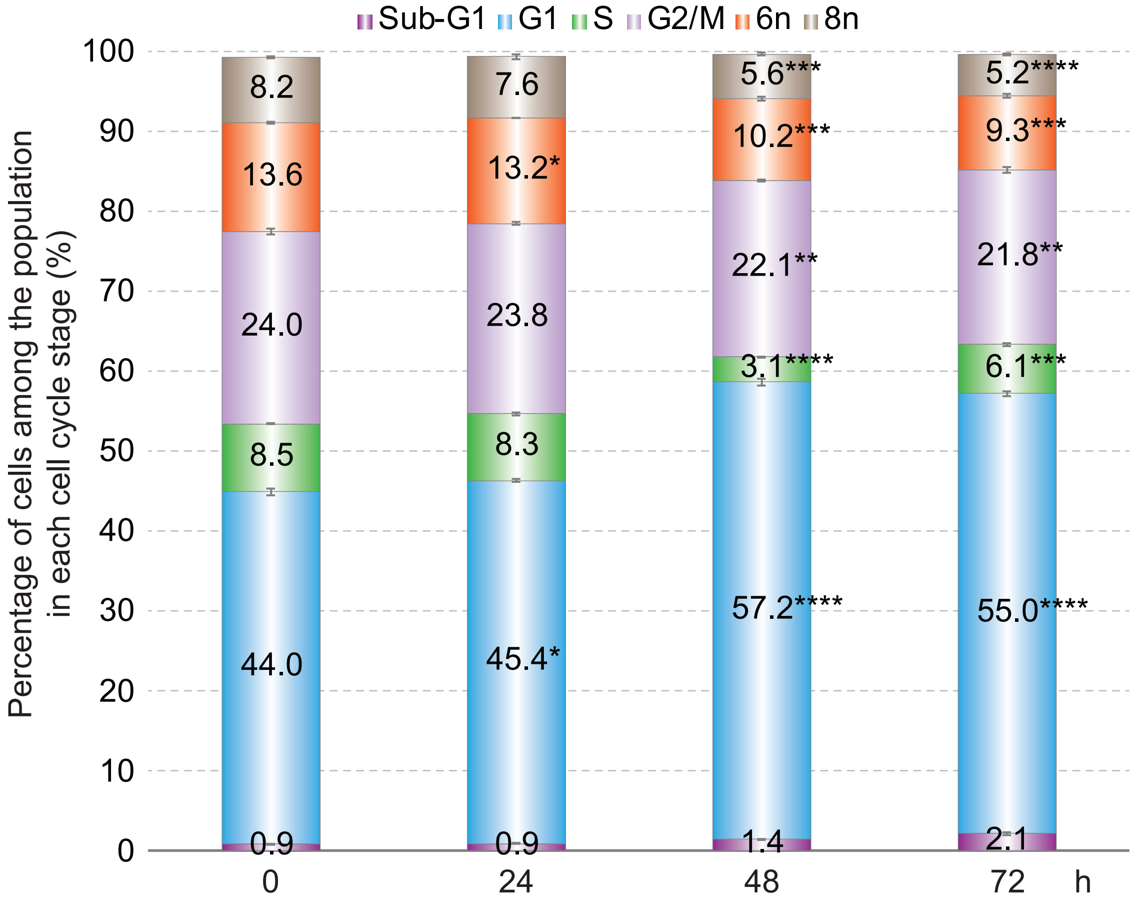

Sayeed et al. Fig. S7

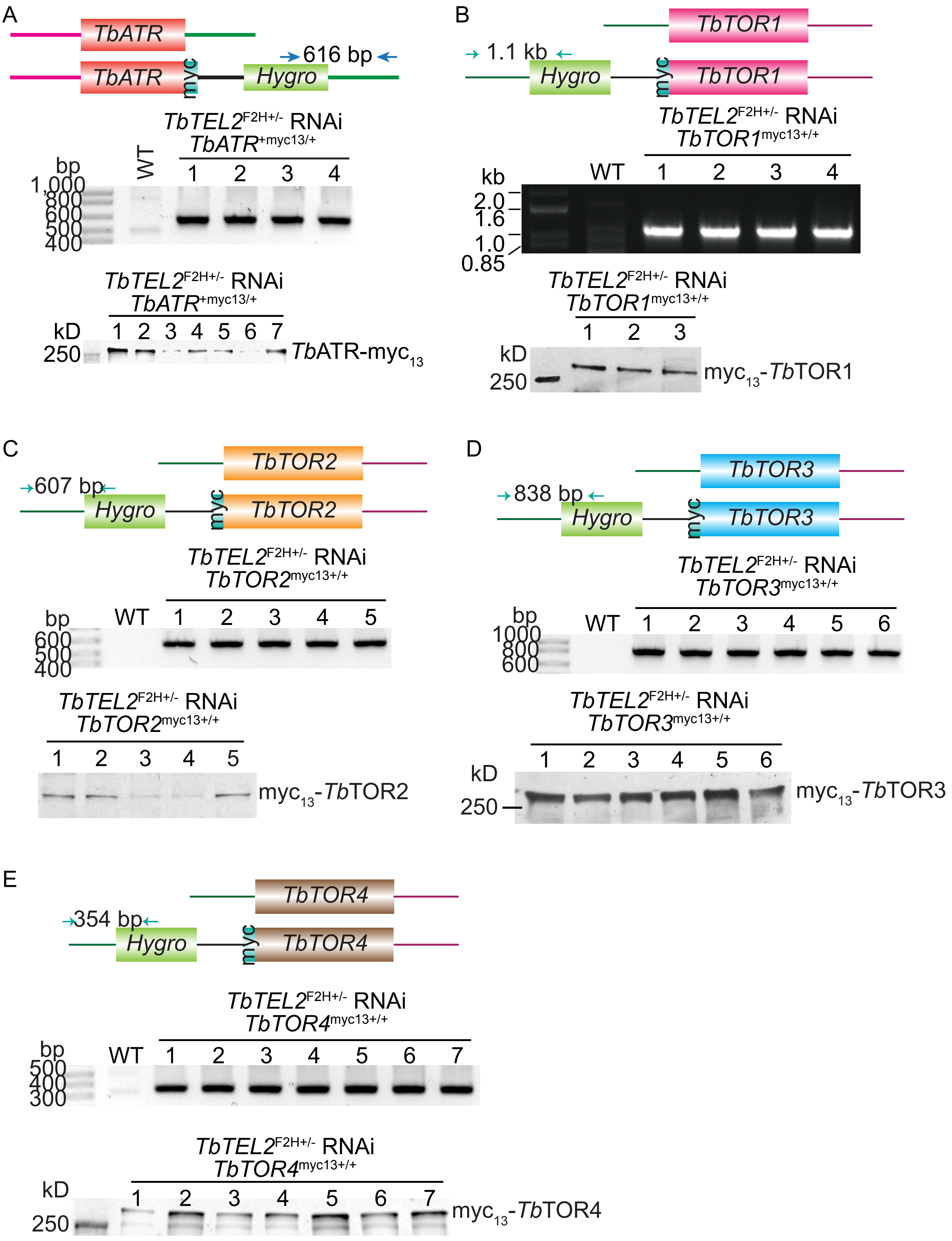

Supplementary References

98. **Kassem, A, Pays, E, Vanhamme, L**. 2014. Transcription is initiated on silent variant surface glycoprotein expression sites despite monoallelic expression in *Trypanosoma brucei*. Proc Natl Acad Sci U S A **111**:8943–8948.
99. **Vanhamme, L, Poelvoorde, P, Pays, A, Tebabi, P, Van Xong, H, Pays, E**. 2000. Differential RNA elongation controls the variant surface glycoprotein gene expression sites of *Trypanosoma brucei*. Mol Microbiol **36**:328–340.
100. **Zhang, Y, Skolnick, J**. 2004. Scoring function for automated assessment of protein structure template quality. Proteins **57**:702–710.
101. **Xu, J, Zhang, Y**. 2010. How significant is a protein structure similarity with TM-score = 0.5? Bioinformatics **26**:889–895.

Supplemental Figure Legends

Figure S1. (A) Sequence alignment of several TEL2 homologs using ClustalW with the slow/accurate pairwise alignment (https://www.genome.jp/tools-bin/clustalw). The amino acid position at the end of each line is indicated on the right. Residues identical to the consensus sequence are highlighted in blue. *Tc*TEL2, *Trypanosoma cruzi* TEL2 (V5BAN0); *Tb*TEL2, *Trypanosoma brucei* TEL2 (Q57VF5); *Lm*TEL2, *Leishmania major* TEL2 (E9AER2); *Hs*TEL2, *Homo sapiens* TEL2 (Q9Y4R8); *At*TEL2, *Arabidopsis thaliana* TEL2 (A0A654FDZ3); *Sc*TEL2, *Saccharomyces cerevisiae* TEL2 (P53038); *Sp*TEL2, *Schizosaccharomyces pombe* TEL2 (Q9P3W5); *Ce*TEL2, *Caenorhabditis elegans* TEL2 (also known as CLK2, Q95YE9). Uniprot protein IDs are listed in the parentheses. Bottom right inset, maximum likelihood phylogenic tree of TEL2 homologs inferred using PhyML. The branch length (in expected substitutions per amino acid position) and the branch support value (in brackets) are indicated near each internal node. Branch length is indicated in brackets after each TEL2 homolog. (B - D) Alignment of AlphaFold 3-predicted structures of TEL2 homologs. (B) Full-length (FL), NT, and CT *Tb*TEL2 structures were separately predicted by AlphaFold 3. The FL *Tb*TEL2 structure was then aligned with the NT *Tb*TEL2 structure (left) or the CT *Tb*TEL2 structure (right). (C) FL, NT, and CT *Hs*TEL2 structures were separately predicted by AlphaFold 3. The FL *Hs*TEL2 structure was then aligned with the NT *Hs*TEL2 structure (left) or the CT *Hs*TEL2 structure (right). (D) The AlphaFold 3-predicted structures of NT domains (left) or CT domains (right) of seven TEL2 homologs and the X-ray structure of *Sc*TEL2 (PDB ID: 3O4Z) (51) are aligned using ChimeraX. The pTM values of TEL2 homologs' predicted structures are listed in Table S1. (E) Sequence alignment of several vertebrate, yeast, and kinetoplastic TEL2 homologs around the CK2-phosphorylation sites (in red boxes) by ClustalW using the alignment reported in (52) as a guide. The amino acid position at the end of each line is indicated on the right.

Figure S2. PCR validation of *TbTEL2*$^{+/-}$ and *TbTEL2*$^{F2H+/-}$ genotypes. (A) A diagram of three *TbTEL2* alleles: N-terminal F2H-tagged, WT, and deleted with the *BSD* marker. Primer locations and expected PCR product sizes are marked. (B) Results of PCR reactions with various primer pairs using gDNA from different *TbTEL2* strains are shown. *TbTEL2* genotypes are shown on the top, primers used are listed below the gel images. Asterisk in the bottom gel represents a non-specific PCR product. (C) Western blotting using an HA monoclonal antibody (HA-probe, Santa Cruz Biotechnology) validated the expression of F2H-*Tb*TEL2. (D) Growth curves of cells with three different genotypes: *TbTEL2*$^{+/+}$, *TbTEL2*$^{F2H+/-}$, and *TbTEL2*$^{+/-}$. Error bars represent standard deviation.

Figure S3. Shorter telomere fragments have a faster growth rate during *T. brucei* cell growth. (A) Southern blotting of AluI and MboI digested genomic DNA isolated from WT cells at various time points using an 800 bp TTAGGG repeat probe. Results of two independent experiments are shown. Sizes of fourteen different telomere bands (marked by arrow heads and dots) were calculated at various time points. (B) Telomere growth rate vs. telomere band size is plotted based on quantified data from (A). The trendline and its formula are shown. (C) Telomere Southern of AluI and MboI digested genomic DNA isolated from GFP-*Tb*TEL2 C2 cells cultured with and without Dox at various time points. Size changes of multiple telomere bands (marked by arrow heads and dots) were quantified. (D) Telomere growth rate vs. telomere band size is plotted for both induced and un-induced C2 cells. The trendlines for both groups of data are shown as dotted lines.

Figure S4. *Tb*TEL2 is not a core component of the *T. brucei* telomere protein complex. (A) IP F2H-*Tb*TEL2 using a mouse HA antibody did not pull down *Tb*RAP1 (detected by a rabbit anti-*Tb*RAP1 antibody). (B & C) Inserting an N-terminal tag to one endogenous *TbTEL2* allele. The diagram of the tagged *TbTEL2* allele is shown at the top, and the PCR result is shown at the bottom in (B). Western blotting was done using a Ty1 mouse antibody in (C). (D & E) Inserting a C-terminal tag to one endogenous *TbTIF2* allele. The diagram of the tagged *TbTIF2* allele is shown at the top, and the PCR result is shown at the bottom in (D). Western blotting was done using an HA mouse antibody in (E). (F) Western blotting shows that *Tb*TRF protein level is not changed upon *Tb*TEL2 depletion.

Figure S5. (A) Principle of the VSG switching assay (75). The parent strain (top left, named S) has a *BSD* marker immediately downstream of the active ES promoter and a *PUR-TK* marker between the active *VSG2* gene and the 70 bp repeats. Before switching, these cells are sensitive to GCV (due to expression of TK) but resistant to puromycin and 100 μg/ml blasticidin. Both *VSG2* and *BSD* genes are intact. *In situ* switching (bottom left) results in silencing of the *VSG2*-resident ES without any genetic rearrangements (so *VSG2* and *BSD* genes should be intact). These cells are sensitive to puromycin and 100 μg/ml blasticidin but resistant to GCV (as TK is silent) and 5 μg/ml blasticidin as a low level of expression can be detected immediately downstream silent ES promotors (98, 99). Switchers arose from Cross-over (top right) should still carry intact *VSG2* and *BSD* genes and resistant to GCV and 100 μg/ml blasticidin. Gene conversion events (middle right) will lead to the loss of the *VSG2* gene alone (in VSG GC switchers) or both *VSG2* and *BSD* genes (in ES GC switchers). The *BSD* gene should be intact in VSG GC switchers, so the cells are expected to be resistant to 100 μg/ml blasticidin. ES GC leads to the loss of the *BSD* gene so the cells will be sensitive to even 5 μg/ml blasticidin. It is possible that some switchers will arise

through ES loss plus *in situ* switch (bottom right), the resulting switchers will have the same phenotype and genotype as ES GC switchers. (B, C) PCR validation of the *TbTEL2*$^{+/-}$ genotype in the S/*Tb*TEL2-SKOi cells. One endogenous *TbTEL2* allele in the S strain was replaced with the hygromycin resistance marker (*HYG*), and the *Tb*TEL2 RNAi construct was targeted to an rRNA spacer subsequently to generate S/*Tb*TEL2-SKOi (Table S3). (B) A diagram of *TbTEL2* alleles in S/*Tb*TEL2-SKOi cells. (C) Results of PCR reactions using P1 & P2 primers or P3 & P4 primers. (D) Western blotting using a rabbit antibody against *T. brucei* RAD51 and TAT-1 against Tubulin (as a loading control). Relative levels of the RAD51 protein were quantified with normalization to the Tubulin levels (indicated at the bottom of the RAD51 western gel). (E) PFGE of undigested genomic DNA from *TbTEL2*$^{F2H+/-}$ RNAi cells induced for 0, 24, 48, and 72 hrs. Most individual megabase chromosomes (> 1 mbp) are discernable in 0- and 24-hr samples but not in 48- and 72-hr samples.

Figure. S6. FACS analysis of *TbTEL2*$^{F2H+/-}$ RNAi cells before and after inducing *Tb*TEL2 RNAi. (A) Cell cycle profiles of *TbTEL2*$^{F2H+/-}$ RNAi cells induced for 0, 24, 48, and 72 hrs. Three independently induced samples were analyzed at each time point. Gates are marked for the first 0-hr sample. All samples were gated the same way. (B) Quantification of percentage of cells at various cell cycle stages among the whole cell population. Average was calculated from three samples and plotted. Error bars represent standard deviation. Unpaired student t-tests were performed to compare percentage of cells at the same cell cycle stage between induced samples (at 24, 48, and 72 hrs) and uninduced samples (at 0 hr). *, $0.05>p>0.01$; **, $0.01>p>0.001$; ***, $0.001>p>0.0001$; ****, $p<0.0001$.

Figure S7. One endogenous allele of each PIKK genes [*TbATR* (A), *TbTOR1* (B), *TbTOR2*(C), *TbTOR3* (D), and *TbTOR4* (E)] was tagged with 13 repeats of myc in *TbTEL2*$^{F2H+/-}$ RNAi cells. In each panel, a diagram of the *PIKK* gene is shown at the top, PCR results validating the targeted tag are shown in the middle, and western results indicating the expression of the tagged PIKK protein are shown at the bottom. Numbers on top of the gel indicate different clone.

Table S1. pTM scores of predicted TEL2 homolog structures

**Protein**	**pTM**
*Tb*TEL2-NT	0.85
*Lm*TEL2-NT	0.86
*Tc*TEL2-NT	0.86
*Hs*TEL2-NT	0.91
*At*TEL2-NT	0.75
*Sp*TEL2-NT	0.89
*Ce*TEL2-NT	0.87
*Tb*TEL2-CT	0.79
*Lm*TEL2-CT	0.78
*Tc*TEL2-CT	0.74
*Hs*TEL2-CT	0.81
*At*TEL2-CT	0.71
*Sp*TEL2-CT	0.8
*Ce*TEL2-CT	0.81

pTM scores: the predicted template modeling (pTM) score is derived from a measure called the template modeling (TM) score. This measures the accuracy of the entire structure (100, 101). A pTM score above 0.5 means the overall predicted fold for the complex might be similar to the true structure.

Table S2. ChimeraX structure alignment parameters (for alignments shown in Fig. 1).

Figure	Panel	Reference protein	Aligned protein	alignment score	# of pruned atom pairs	RMSD (Å) for pruned atom pairs	# of all atom pairs	RMSD (Å) for all atom pairs
1	C	*Tb*TEL2-NT	*Tc*TEL2-NT	1154.2	309	1.092	458	4.157
		*Tb*TEL2-NT	*Lm*TEL2-NT	788.6	231	1.056	423	4.029
		*Tb*TEL2-CT	*Tc*TEL2-CT	1305.8	298	0.853	405	9.116
		*Tb*TEL2-CT	*Lm*TEL2-CT	957	265	0.954	382	11.587
1	D	*Tb*TEL2-NT	*Hs*TEL2-NT	314.8	56	1.049	388	19.306
		*Tb*TEL2-CT	*Hs*TEL2-CT	333.8	123	1.16	310	18.272
1	E	*Tb*TEL2-NT	*Sc*TEL2-NT	258.8	13	1.615	320	17.769
		*Tb*TEL2-CT	*Sc*TEL2-CT	256.9	71	1.028	243	15.023

Table S3. List of Strains and Plasmids

Parent strain	Purpose	Transfected plasmid	RE Digestion	Marker	Resulting strain
WT (SM)	To delete one *TbTEL2* allele	pSK-*Tb*TEL2-SKO-BSD	XhoI & NotI	BSD	*TbTEL2*$^{+/-}$
WT (SM)	To tag one *TbTEL2* allele with an N-terminal FLAG-HA-HA (F2H) epitope	pSK-Puro-F2H-*Tb*TEL2-New	ApaI & NotI	PUR	*TbTEL2*$^{F2H+/+}$
TbTEL2$^{+/-}$	To tag one *TbTEL2* allele with an N-terminal F2H epitope	pSK-Puro-F2H-*Tb*TEL2-New	ApaI & NotI	PUR	*TbTEL2*$^{F2H+/-}$
TbTEL2$^{F2H+/-}$	To introduce a *Tb*TEL2 RNAi construct	pZJMβ-*Tb*TEL2-RNAi-1/2	NotI	BLE	*TbTEL2*$^{F2H+/-}$ RNAi
S (HSTB261)	To delete one *TbTEL2* allele	pSK-*Tb*TEL2-SKO-Hygro	XhoI & NotI	HYG	S/*Tb*TEL2 SKO
S (HSTB261)	To establish a control strain for the VSG switching assay	pZJMβ	NotI	BLE	S/ev
S/*Tb*TEL2 SKO	To introduce a *Tb*TEL2 RNAi construct	pZJMβ-*Tb*TEL2-RNAi3/2	NotI	BLE	S/*Tb*TEL2-SKOi
WT (SM)	To tag one *TbTEL2* allele with an N-terminal Ty1 epitope	pSK-Ty1-*Tb*TEL2-target	ApaI & NotI	BLE	*TbTEL2*$^{Ty1+/+}$
TbTEL2$^{Ty1+/+}$	To tag one *TbTIF2* allele with a C-terminal F2H epitope	PSK-*Tb*TIF2-F2H-α/β-Puro-3'UTR-Tar	ApaI & NotI	PUR	*TbTEL2*$^{Ty1+/+}$ *TbTIF2*$^{+F2H/+}$
TbTEL2$^{F2H+/-}$ RNAi	To tag one *TbTOR1* allele with an N-terminal 13 x myc (myc$_{13}$) epitope	pSK-13XMyc-*Tb*TOR1-Hygro-α/β-tubinter	PvuII & NotI	HYG	*TbTEL2*$^{F2H+/-}$ RNAi *TbTOR1*$^{myc13+/+}$
TbTEL2$^{F2H+/-}$ RNAi	To tag one *TbTOR2* allele with an N-terminal myc$_{13}$ epitope	pSK-13XMyc-*Tb*TOR2-Hygro-α/β-tubinter	BsmI & NotI	HYG	*TbTEL2*$^{F2H+/-}$ RNAi *TbTOR2*$^{myc13+/+}$
TbTEL2$^{F2H+/-}$ RNAi	To tag one *TbTOR3* allele with an N-terminal myc$_{13}$ epitope	pSK-13XMyc-*Tb*TOR3-Hygro-α/β-tubinter	AfeI & NotI	HYG	*TbTEL2*$^{F2H+/-}$ RNAi *TbTOR3*$^{myc13+/+}$
TbTEL2$^{F2H+/-}$ RNAi	To tag one *TbTOR4* allele with an N-terminal myc$_{13}$ epitope	pSK-13XMyc-*Tb*TOR4-Hygro-α/β-tubinter	EarI & NotI	HYG	*TbTEL2*$^{F2H+/-}$ RNAi *TbTOR4*$^{myc13+/+}$
TbTEL2$^{F2H+/-}$ RNAi	To tag one *TbATR* allele with a C-terminal myc$_{13}$ epitope	pSK-*Tb*ATR-13XMyc-α/β-tubinter-Hygro	ApaI & NotI	HYG	*TbTEL2*$^{F2H+/-}$ RNAi *TbATR*$^{+myc13/+}$
WT (SM)	To introduce a GFP-*Tb*TEL2 conditional expression construct	pCO57-nG-*Tb*TEL2	NotI	BLE	GFP-*Tb*TEL2

Table S7. List of qRT-PCR primer sequences

Primer Name	Sequence (5' - 3')
OBL-TOR1-RT-FW	GATGTGGAGTATGAGGCCATC
OBL-TOR1-RT-BW	ATGAAGACATTCGGCAACGACG
OBL-TOR2-RT-FW	GATGTAGTTGAGGGTGGTACAG
OBL-TOR2-RT-BW	ACCTCCTGTAGCATCAACAAGG
OBL-TOR3-RT-FW	ACCCACACGAATTGAGGTGTAG
OBL-TOR3-RT-BW	TGTATGAGACAAGGTGGCGAG
OBL-TOR4-RT-FW	GTTGTCAACATCGGCGGAG
OBL-TOR4-RT-BW	TGTCGTCGACAGGCTCATC
OBL-ATR-RT-FW	GTTGCAGCTGGTGCAGAC
OBL-ATR-RT-BW	TATCGCCTATGGCGCATGTC
OBL-VSG221-FW1	GTCCTAGCCCAAGTTCTTC
OBL-VSG221-BW1	GCTGTTGCAGTAGCTGTTAC
OBL-VSG224-RT-FW	CAGTCTTGTGCGCACTAGCT
OBL-VSG224-RT-BW	ATGCTGCTGCTGCTGTTACC
OBL-VSGVO2-RT-FW	AGCTTATCTAGCAGACGCCG
OBL-VSGVO2-RT-BW	TCCTCCCATTTCCTCCGATC
OBL-VSG16-FW	CGGATCAAAGATAGCAGGGC
OBL-VSG16-probe-BW	GCCAGTGAACATACCTGTCG
OBL-mVSG397-FW	GATACCAAGCAGTACTGGCC
OBL-mVSG397-RT-BW	GCTTGTGCCTGTTGTTCCAG
OBL-mVSG531-FW	GGCTCACATTCTCTGAGGTG
OBL-mVSG531-RT-BW	TTCCTTCTTGAGCGCTGCG
OBL-mVSG653-FW	TTGCCGCTTACGCTAGCG
OBL-mVSG653-RT-BW	CCTTAGAAGGTTGACTGTCGC
OBL-bTubulin-RT-FW	ATCTTTGGACAGTCTGGCGC
OBL-bTubulin-RT-BW	TTCCACAAGTTGGTGCACGG
OBL-rDNA-RTPCR-1	ACGGAATGGCACCACAAGAC
OBL-rDNA-RTPCR-2	GTCCGTTGACGGAATCAACC
OBL-tbTERT-RT-FW1	ATCGGTTTGCAGCTGCGTTG
OBL-tbTERT-RT-BW1	TGCGGATGGTCTCCATCCAT
SNAP50-RT-FW2	CTATGAAGATTTGACGGCACCC
SNAP50-RT-BW2	CTCGTGTGGTCATCTCGATCAA

Table S8. List of primers used in LMPCR

Name	Sequence	Purpose
LMPCR-Linker-short	GTGAATTCAGATC	for making LMPCR adaptor
LMPCR-Linker-long	GCGGTGACCCGGGAGATCTGAATTCAC	for making LMPCR adaptor
OBL-SNAP50-oligo-probe_NT	CAATTGGTGAGGCAGAGGATGCGATG	Southern hybridization probe
OBL-70bp-oligo-probe-new	GGAGAGTGTTGTGAGTGTGTGTATATACG AATATTATAATAAG	Southern hybridization probe
OBL-VSG21-oligo-probe-revised	AACAAACTACGCGTTGTAGCATCTTCTAA AACAGGTGGGGCG	Southern hybridization probe
OBL-VSG2-oligo probe-revised	GGTGCGTTGTTTACGCTGCAAGCAGCGGC GAGCAAAATCCAGAAAATG	Southern hybridization probe
OBL-Tb427-090056400-FW	CCCACAACTTGATGTCTACAAAATTAG	Locus-specific primer for PCR reaction
OBL-70bp-BES40-new-FW	GTGTTATAGTCGTAATAGAAGCTGAAG	Locus-specific primer for PCR reaction
OBL-VSG21-new-FW	CAAGTCATTACATGCGTCACATAAC	Locus-specific primer for PCR reaction
OBL-VSG2-LMPCR-new-FW	CCAAGTTCTTCCAATTCTTGTCG	Locus-specific primer for PCR reaction
OBL-TbRAP1-NT-78aa-FW	GTGATTGCACCGTGGAAACG	For loading control PCR
OBL-TbRAP1-NT-188aa-BW	ACACGAAGAAATCGGGACGTG	For loading control PCR
