# Supplementary material for: Trypanosoma brucei TEL2 inhibits VSG switching and protects PIKKs from the 26S proteasome-mediated degradation
Source: Microbiol Spectr. 2026 May 29;14(7):e03516-25. doi: 10.1128/spectrum.03516-25 (PMC13340017; doi:10.1128/spectrum.03516-25)
Supplement: Reviewer comments [file reviewer-comments.pdf]

AMERICAN SOCIETY FOR MICROBIOLOGY | Microbiology Spectrum

# *Trypanosoma brucei* TEL2 inhibits VSG switching and protects PIKKs from the 26S proteasome-mediated degradation

SK Sayeed, Prem Kushwaha, Marjia Afrin, and Bibo Li

*Corresponding Author(s): Bibo Li, Cleveland State University*

**Review Timeline:**

| | |
|---|---|
| Submission Date: | October 29, 2025 |
| Editorial Decision: | December 29, 2025 |
| Revision Received: | March 10, 2026 |
| Accepted: | April 22, 2026 |

*Editor: Galadriel Hovel-Miner*

*Reviewer(s): Disclosure of reviewer identity is with reference to reviewer comments included in decision letter(s). The following individuals involved in review of your submission have agreed to reveal their identity: Mark Ewusi Shiburah (Reviewer #1)*

## Transaction Report:

(Note: With the exception of the correction of typographical or spelling errors that could be a source of ambiguity, letters and reports are not edited. The original formatting of letters and referee reports may not be reflected in this compilation.)

**DOI: https://doi.org/10.1128/spectrum.03516-25**

Re: Spectrum03516-25 (***Trypanosoma brucei* TEL2 inhibits VSG switching and protects PIKKs from the 26S proteasome-mediated protein degradation**)

Dear Dr. Bibo Li:

Thank you for the privilege of reviewing your work. Below you will find my comments, instructions from the Spectrum editorial office, and the reviewer comments.

Please return the manuscript within 60 days; if you cannot complete the modification within this time period, please contact me. If you do not wish to modify the manuscript and prefer to submit it to another journal, notify me immediately so that the manuscript may be formally withdrawn from consideration by Spectrum.

**Revision Guidelines**
To submit your modified manuscript, log into the submission site at https://spectrum.msubmit.net/cgi-bin/main.plex. Go to Author Tasks and click the appropriate manuscript title to begin. The information you entered when you first submitted the paper will be displayed; update this as necessary. Note the following requirements:

• Upload point-by-point responses to the issues raised by the reviewers in a file named "Response to Reviewers," NOT in your cover letter.
• Upload a compare copy of the manuscript (without figures) as a "Marked-Up Manuscript" file.
• Upload a clean .DOC/.DOCX version of the revised manuscript and remove the previous version.
• Each figure must be uploaded as a separate, editable, high-resolution file (TIFF or EPS preferred), and any multipanel figures must be assembled into one file.
• Any supplemental material intended for posting by ASM should be uploaded with their legends separate from the main manuscript. You can combine all supplemental material into one file (preferred) or split it into a maximum of 10 files with all associated legends included.

For complete guidelines on revision requirements, see our Submission and Review Process webpage. Submission of a paper that does not conform to guidelines may delay acceptance of your manuscript.

**Data availability:** ASM policy requires that data be available to the public upon online posting of the article, so please verify all links to sequence records, if present, and make sure that each number retrieves the full record of the data. If a new accession number is not linked or a link is broken, provide Spectrum production staff with the correct URL for the record. If the accession numbers for new data are not publicly accessible before the expected online posting of the article, publication may be delayed; please contact production staff (Spectrum@asmusa.org) immediately with the expected release date.

**Publication Fees:** For information on publication fees and which article types are subject to charges, visit our website. If your manuscript is accepted for publication and any fees apply, you will be contacted separately about payment during the production process; please follow the instructions in that e-mail. Arrangements for payment must be made before your article is published.

**ASM Membership:** Corresponding authors may join or renew ASM membership to obtain discounts on publication fees. Need to upgrade your membership level? Please contact Customer Service at Service@asmusa.org.

The ASM Journals program strives for constant improvement in our submission and publication process. Please tell us how we can improve your experience by taking this quick Author Survey.

Thank you for submitting your paper to Spectrum.

Sincerely,
Galadriel Hovel-Miner
Editor
Microbiology Spectrum

Reviewer #1 (Public repository details (Required)):

The data on RNA Levels represented in the graphs of Figs 3, 5F, must be deposited

Reviewer #1 (Comments for the Author):

1. A section on the type of statistical tests used in the analysis must be provided to help readers.

2. Does the parasite eventually die upon TEL 2 depletion? A decline is observed in the growth curve for Fig. 2B after some days, but no comments on the survivability are made

3. In relation to lines 180-187: There are previous reports of ALT activity in trypanosomes. Could this be why we do not see strong effects on telomeres? Additionally, the comparison for shortened telomere will be best judged by comparing within-strain differences at the different PDs

4. In relation to line 187: Given the challenge with growth when induced, the population doubling will take longer -the authors may want to calculate the population doubling time for the TbTEL2RNAi and factor this into the expectation for the telomere length

Additionally, I suppose that for an ordinary agarose gel Southern, the telomere sizes will be difficult to differentiate if the variation is only 200 bp. An additional quantitative approach, such as FlowFISH or sequencing, can provide a different picture.

5. Grammar: Line 239, Change commence to commencement

6. Line 275, what could account for this anomaly? The expectation of an increase in TERRA should correlate with an increase in DNA damage signals, as it activates DDR pathways. Is there a possibility that some DNA damage repair mechanisms work more efficiently to resolve the damage? The kinase argument is equally valid; however, considering the early results of H2A phosphorylation, the idea of an efficient DDR response to resolve the signals is tenable. The alternate pathway presented in the discussion is also a valid argument.

7. Given that ATM is highly related to H2A kinase activity, it would have been useful to show the regulation of this protein by TEL2 through alternative assays to measure the ATM activity, since tagging failed. Also, a complementary assay using Rad51 to check for DNA damage can offer some insights into the mechanistic DNA damage

8. Additionally, some data on the cell cycle could help tie the results to the growth phenotype

9. A lot of experiments were performed, but the reasoning behind the experiments was not explained

Reviewer #2 (Comments for the Author):

See attachment

Reviewer #3 (Comments for the Author):

This manuscript by Sayeed et al. identifies the Tel2 protein of T. brucei and begins to characterize some of its functions. The manuscript is clearly written and straightforward with the results shown justifying the conclusions drawn. The work described is a modest but important step forward in the field. I have only minimal criticisms, listed below.

Is there data on whether the GFB-TEL2 fusion is functional, having the essential TEL2 function? Or, is this just assumed in the ectopic expression work?

Does the level of expression of the fusion protein change over the growth time course? This might be worth knowing given the bit of change in growth occurring early on.

In this study Sayeed et al identified a structural homolog for TEL2, a protein first identified in yeast as a regulator of telomere size but subsequently shown to play a central role in PIK kinase protein stability in multiple pathways in organisms ranging from yeasts to humans. The TbTEL2 homolog shows only weak protein sequence homology with other TEL2 species but shares strong protein motif conservation and, using the predictive AI, AlphaFold 3, a high level of both N-terminal and C-terminal module structural similarities. An analysis of telomere tract sizes by restriction analysis revealed no detectable changes in size after TEL2 depletion and after TEL2 overproduction and no stable association of TEL2 with telomere proteins. The authors present data demonstrating that depletion of TEL2 results in both a loss of monoallelic VSG expression and a significant increase in the rate of switching at multiple sites as well, Numerous additional sources of genomic instability were also identified after TEL2 depletion. The investigators use ligation mediated assay to detect detection genomic DNA breaks after TEL2 downregulation. Using tagged PIKKs, ATR, TOR2, TOR1, TOR3, and TOR4, only ATR showed association with Tel2 by IP. They further demonstrated that PIKKs were depleted under conditions of TEL2 depletion suggesting a role of TEL2 in maintaining the stability of PIKKS. Arrest by cycloheximide demonstrated that this effect was post translational. Interestingly, the proteosome inhibitor MG132 reversed some or all the depletion caused TEL2 loss. The authors propose that T. brucei uses a mechanism different than other TEL2 homologs to influence PIKK abundance that are mediated through an Hsp90-chaperone mechanism.

This is an interesting and significant study on the first T. brucei TEL2 homolog. The manuscript is  strongest in its demonstration that TEL2 is involved in the regulation of VSG switching and in PIKKs stability. The study suffers however from experimental, interpretive, and conceptual limitations that need to be addressed before publication.

**Major Issues**

*Role of TEL2 in a unique mechanism of PIKK stability.*

1. The authors present an interesting finding that, unlike other TEL2 homologs, the T. brucei protein is mediated by the 26S proteosome. The data presented are difficult to interpret since it is unclear whether the proteosome mechanism explains most or all of PIKK species, as a statistically vali quantitative analysis is not presented. This leaves open the possibility that the proteosome mechanism is present in addition to the Hsp90 mechanism.
2. Part of this reviewer’s skepticism is that the structurally similar TEL2 of budding yeast associates with the Tel2-Tti1-Tti2 complex (Hoffman et al 2016). This raises the obvious question as to whether the T. brucei protein can also form this complex given the level of structural conservation. For a definitive conclusion, this possibility needs to be investigated directly. Alternatively, the authors can limit their conclusion that the TbTel2-mediated PIKK stability is solely mediated through a proteasomal mechanism.
3. Given their hypothesis the authors need to discuss how they envision such a structurally similar protein to be mediated by a distinct mechanism. Do the authors consider this to be a separate kinetoplast branch of function?

*Is there a DDR in PIKK depleted cells?*

The authors claim that the absence of sustained gamma H2AX indicates the absence of a DNA damage response pathway. At this same time, they indicate that histone kinases are depleted after TbTEL2 inhibition. If this is the case, how can the absence of the signal indicate the absence of a DDR. This is a point of confusion that needs to be addressed.

*Limitations in Sensitivity*

The telomere data only shows that there are no large-scale changes in telomere dynamics. However, the assays used are not sensitive enough to draw a definitive conclusion. The absence of a means to detect a single telomere in T. brucei by a STELA like assay is a limitation in this regard. However, the possible improvement in this analysis is suggested by the violin plots after overproduction of TbTEL2. There is some indication that the fraction of *shortest* telomeres is increased. It may well be a mistake to look for changes in the mean distribution. In this regard, is the increased fraction of shorter telomeres reproducible?

The IP studies rule out, as the authors point out, a stable association of TbTEL2 with other telomeric proteins. However, in context of chromatin, this is a rather insensitive assay. The authors should reproduce their PICH assay with the overproduced tagged TEL2 species. This would be a more critical test of the association with Tel2 with other telomere proteins.

The association of TbTEL2 by IP with ATR is important. It is unfortunate that ATM association could not be tested. Have the authors tried FLAG tagging of ATM. This has often led to the production of active protein. Could the authors state the technical limitation of tagging TEL2 in Tb. Given that Tel2 has been demonstrated to associate with ATM in yeast, this is an important omission.

*Interpretation of LMPCR*

Some verification of the presence of DNA breaks would be preferable given the heterogenous set of breaks produced by the I LMPCR assay. As a control, conduct an experiment in which the end structure is resected making breaks unable to ligate to ensure the absence of any species

Another approach to test for the validity of the LMPCR is using CHEF gene analysis to detect breaks directly (as well as other forms of recombination that is occurring).

**Textual Issues**

Line 75. Explain why an analysis of telomeres is central to understanding VSG switching. This is a critical issue that is omitted in the Introduction.

Line 261: The authors demonstrate in Figure 3 the presence of other classes of genetic change but do not present any primary data substantiating these rearrangements. How

were the different classes defined? There is also no statistical analysis of the differences in the presence and absence of Tel2.

Line 318: The level of each transcript is given in Figure 5. However, what were the number of repetitions and variations between repeats?

Line 323. The absence of a major shift in RNAs show that PIKK levels are maintained by a post-transcriptional mechanism (not post-translational) as stated. Although unlikely, the authors do not discuss the possibility of reason for ruling out a translational mechanism.

Line 342. The authors show that in all cases except for TOR3, MG132 reverses the depletion. However, these analyses seem to be qualitative so that it is difficult to ascertain whether the reversal accounts for all or part of the TbTEL2-mediated PIKK loss of stability.

Line 363: Although the authors show the kinetoplastid TEL2 homologs are most conserved, the functional significance of this is unclear since this represents the first functional analysis.

Line 392. Please explain types of DDR pathways independent of gamma H2A.

Line 460: VSG Switching Assay: The description of this assay In the Materials and Methods does not seem to match that in the text. The text states that selection for VSG2 is released and switching is analyzed. Please clarify.

Line 474: What do you mean by “VSG2 rabbit antibody (without the cross-reaction proportion)”?

**Figure Related Issues**

Figure 1:

A. Provide a brief description of the functional significance of the TEL2 arm and telomere length regulation protein motifs.
B. Explain the meaning of the color coded LDDT values.

C-E, provide statistical power of comparisons associated with each of these comparisons.

Figure 2 (all). Please present panels in order of presentation

Figure 2F.

Are the species denoted by the arrows based on a previous analysis that verified their identity of single species increasing in size?

Figure 2G.

Is the lower bound of the GFP-TbTel2 reproducible in violin plots? How much overproduction is achieved with the GFP proteins?

Figure 3 (all) Place the panels in order of presentation

Figure 3A> The error bars for fold changes in RNA levels are quite large. What is the reason for this?

Figure 6. Present the aggregate data for all the repeats and quantification of data.

Supplementary Figure 1

The legends indicates that the sequences were compared to the consensus sequence. There is no TEL2 consensus. Please explain.

Supplementary Figure 4

What do the numbers above each lane refer to?

The myc tagged species appear to be at the limit mobility of the gel in A, and E. A better separation is needed for the identification.

This manuscript by Sayeed et al. identifies the Tel2 protein of T. brucei and begins to characterize some of its functions. The manuscript is clearly written and straightforward with the results shown justifying the conclusions drawn. The work described is a modest but important step forward in the field. I have only minimal criticisms, listed below.

Is there data on whether the GFB-TEL2 fusion is functional, having the essential TEL2 function? Or, is this just assumed in the ectopic expression work?

Does the level of expression of the fusion protein change over the growth time course? This might be worth knowing given the bit of change in growth occurring early on.

RE: Spectrum03516-25

Galadriel Hovel-Miner
Editor
Microbiology Spectrum

March 7, 2026

Dear Dr. Hovel-Miner,

We would like to thank you and the reviewers for reviewing our manuscripts carefully and for providing us constructive suggestions. We are grateful that the reviewers consider our work "*an interesting and significant study"* and regard our manuscript *"clearly written and straightforward with the results shown justifying the conclusions drawn".* We have carefully reviewed the comments and revised our manuscript accordingly. Below please find our point-by-point responses to all reviewers. The reviewers' critiques are in the *italic Palatino* font, while our responses are in the regular Arial font.

Thank you!

Bibo Li

*Reviewer #1 (Public repository details (Required)):*

*The data on RNA Levels represented in the graphs of Figs 3, 5F, must be deposited.*

We thank the reviewer for this reminder. We have now included raw data for the qRT-PCR experiments in Table S4 and added more qRT-PCR experiment details in Methods. We have already included qRT-PCR primers in Table S7. All results of these qRT-PCR experiments have been included in the current manuscript.

*Reviewer #1 (Comments for the Author):*

*1. A section on the type of statistical tests used in the analysis must be provided to help readers.*

We thank the reviewer for this suggestion. We have now added a "Statistical analyses" section in Methods (pages 19 – 20, lines 620 – 645). We have indicated in figure legends, wherever applicable, what error bars represent. We have also provided description about various statistical analyses done when examining telomere fragment sizes and telomere growth rates (page 7, lines 203 – 207; page 8, lines 224 – 230) and when VSG switching patterns were compared between *Tb*TEL2 depleted and control cells (page 10, lines 290 – 295).

*2. Does the parasite eventually die upon TEL 2 depletion? A decline is observed in the growth curve for Fig. 2B after some days, but no comments on the survivability are made.*

We thank the reviewer for this comment. We have stated that "*Tb*TEL2 is essential for cell proliferation" in the text, and we believe *Tb*TEL2 is essential for parasite survival. However, depleting essential gene products by RNAi in *T. brucei* frequently leads to parasite adaptation, so that a subpopulation of the cells can eventually recover. We observed that some *Tb*TEL2 RNAi clones can overcome the growth arrest after 96-hr of RNAi induction. This is why we only examined the growth phenotype within 72 hrs after inducing *Tb*TEL2 RNAi. To be more rigorous, we would only state that "*Tb*TEL2 is essential for *T. brucei* proliferation".

*3. In relation to lines 180-187: There are previous reports of ALT activity in trypanosomes. Could this be why we do not see strong effects on telomeres? Additionally, the comparison for shortened telomere will be best judged by comparing within-strain differences at the different PDs*

We thank the reviewer for this suggestion. In *Leishmania*, deleting TERT (the protein component of telomerase) leads to more telomeric C-circles, suggesting more telomeric recombination, which is similar to what has been observed in mammalian ALT cancer cells (Alpizar-Sosa et al. 2025. BMC Geomics). In *T. brucei*, a typical ALT mechanism has not been reported, except that short silent *VSG*-marked telomeres appear to be stabilized when they are shortened to only 40 bp in telomerase null (TERT null) cells (Dreesen & Cross, 2006. MCB). We have not considered the involvement of ALT in *Tb*TEL2 RNAi cells. First, none of the known TEL2 homologs directly regulates telomerase activity. We have not observed any phenotype suggesting *Tb*TEL2's role in telomerase activity regulation, either. Second, a typical sign for ALT mechanism is telomeric recombination, which can lead to dramatic telomere length changes within just a few PDs. This is why we performed the telomere Southern in *Tb*TEL2 RNAi cells. Even though these cells only grew for just a few PDs, we should be able to see some dramatic telomere length changes if telomere recombination was involved. We did not observe any dramatic telomere length changes, suggesting that ALT is not involved in *Tb*TEL2 depleted cells. We have now added the following text in the manuscript: "Importantly, we did not observe any dramatic telomere length changes that are frequently mediated by telomere recombination (66-68), suggesting that *Tb*TEL2 depletion does not activate a telomerase-independent alternative telomere maintenance mechanism usually depending on telomere recombination, at least within a few days." (Page 7, lines 187 – 190). When examining telomere length change rate, we always calculate the change/PD value.

*4. In relation to line 187: Given the challenge with growth when induced, the population doubling will take longer -the authors may want to calculate the population doubling time for the TbTEL2RNAi and factor this into the expectation for the telomere length. Additionally, I suppose that for an ordinary agarose gel Southern, the telomere sizes will be difficult to differentiate if the variation is only 200 bp. An additional quantitative approach, such as FlowFISH or sequencing, can provide a different picture.*

Indeed, depletion of *Tb*TEL2 leads to a growth arrest. Our observation indicates that *Tb*TEL2 is essential for *T. brucei* proliferation. Therefore, it is impossible to make *Tb*TEL2-depleted cells to grow longer than they can. Nevertheless, we calculated telomere length change per PD as the change rate, which have already taken into consideration of different numbers of PD that these cells can grow before and after *Tb*TEL2 depletion. Telomere Southern is sensitive enough to determine telomere length changes when cells are cultured for a long-term. This method has been routinely used in many previous published papers to examine telomere length. In the current manuscript, Fig. 2F and Fig. S3, A&C all showed that we can determine the telomere growth rate using this approach. In Fig. 2C, we did not observe any discernable telomere length changes because the cells grew for a very short time. We also agree with the reviewer that other tools are available to examine telomere length changes in higher resolution, which can be explored in future *T. brucei* studies.

*5. Grammar: Line 239, Change commence to commencement*

We thank the reviewer for pointing this out. We have corrected it as suggested (page 9, line 267).

*6. Line 275, what could account for this anomaly? The expectation of an increase in TERRA should correlate with an increase in DNA damage signals, as it activates DDR pathways. Is there a possibility that some DNA damage repair mechanisms work more efficiently to resolve the damage? The kinase argument is equally valid; however, considering the early results of H2A phosphorylation, the idea of an efficient DDR response to resolve the signals is tenable. The alternate pathway presented in the discussion is also a valid argument.*

We thank the reviewer for this comment. First, to confirm this observation, we have repeated the experiment again and did a longer exposure for the γH2A western image. This has been added to Fig. 3G. With longer exposure, we can clearly see γH2A signal 24 and 48 hrs after *Tb*TEL2 RNAi induction, but the signal disappeared by 72 hrs after induction. In addition, we have now found that the *Tb*RAD51 level increases upon *Tb*TEL2 depletion by western (Fig. S5D). The *Tb*RAD51 level change pattern is similar to that of γH2A. Importantly, we clearly observed many DNA breaks by LMPCR upon *Tb*TEL2 depletion (Fig. 4). Furthermore, we have now performed PFGE and found that undigested *T. brucei* genomic DNA became more smeared at 48 and 72 hrs after depletion of *Tb*TEL2 (Fig. S5E). Therefore, even though the γH2A and *Tb*RAD51 signaling was only activated transiently, there was clearly more genome wide DNA damage upon *Tb*TEL2 depletion.

Although depletion of *Tb*TEL2 increases the TERRA level, the increase is only 2-fold, and we have discussed that this is unlikely to be the major reason to affect the global genome integrity (page 14, lines 434 – 439).

There is at least one *Tb*ATM/*Tb*ATR-independent DNA damage response pathway (ref 53), and we have added the discussion that *Tb*TEL2 depletion may activate this pathway, which in turn induced more VSG switching (page 16, lines 496 – 498). However, it has not been compared carefully whether different DDR machinery may act with different efficiencies in *T. brucei*. In addition, we have discussed the possibility that both $\gamma$H2A kinases ATM and ATR are depleted in *Tb*TEL2-depleted cells (page 16, lines 484 – 498).

*7. Given that ATM is highly related to H2A kinase activity, it would have been useful to show the regulation of this protein by TEL2 through alternative assays to measure the ATM activity, since tagging failed. Also, a complementary assay using Rad51 to check for DNA damage can offer some insights into the mechanistic DNA damage*

We thank the reviewer for this suggestion. We have tried to tag ATM for an extended length of time (more than 2 years) without being successful. We have now performed western and shown that *Tb*RAD51 level increased upon *Tb*TEL2 depletion (Fig. S5D).

*8. Additionally, some data on the cell cycle could help tie the results to the growth phenotype*

We thank the reviewer for this suggestion. We have now performed FACS analysis on *Tb*TEL2 RNAi cells (Fig. S6). We found that depletion of *Tb*TEL2 leads to an accumulation of G1 cells, which is the same as that observed in *Tb*TOR1-depleted cells. We have discussed this finding in the Discussion section (page 15, lines 453 – 456).

*9. A lot of experiments were performed, but the reasoning behind the experiments was not explained*

We thank the reviewer for this suggestion. We have now revised the manuscript, adding more explanation of the rationales.

*Reviewer #2 (Comments for the Author):*

*In this study Sayeed et al identified a structural homolog for TEL2, a protein first identified in yeast as a regulator of telomere size but subsequently shown to play a central role in PIK kinase protein stability in multiple pathways in organisms ranging from yeasts to humans. The TbTEL2 homolog shows only weak protein sequence homology with other TEL2 species but shares strong protein motif conservation and, using the predictive AI, AlphaFold 3, a high level of both N-terminal and C-terminal module structural similarities. An analysis of telomere tract sizes by restriction analysis revealed no detectable changes in size after TEL2 depletion and after TEL2 overproduction and no stable association of TEL2 with telomere proteins. The authors present data demonstrating that depletion of TEL2 results in both a loss of monoallelic VSG expression and a significant increase in the rate of switching at multiple sites as well, Numerous additional sources of genomic instability were also identified after TEL2 depletion. The*

*investigators use ligation mediated assay to detect genomic DNA breaks after TEL2 downregulation. Using tagged PIKKs, ATR, TOR2, TOR1, TOR3, and TOR4, only ATR showed association with Tel2 by IP. They further demonstrated that PIKKs were depleted under conditions of TEL2 depletion suggesting a role of TEL2 in maintaining the stability of PIKKS. Arrest by cycloheximide demonstrated that this effect was post translational. Interestingly, the proteosome inhibitor MG132 reversed some or all the depletion caused TEL2 loss. The authors propose that T. brucei uses a mechanism different than other TEL2 homologs to influence PIKK abundance that are mediated through an Hsp90-chaperone mechanism. This is an interesting and significant study on the first T. brucei TEL2 homolog. The manuscript is strongest in its demonstration that TEL2 is involved in the regulation of VSG switching and in PIKKs stability. The study suffers however from experimental, interpretive, and conceptual limitations that need to be addressed before publication.*

We thank the reviewer for a careful review. We have now revised the manuscript as suggested.

***Major Issues***

*Role of TEL2 in a unique mechanism of PIKK stability.*

*1.The authors present an interesting finding that, unlike other TEL2 homologs, the T. brucei protein is mediated by the 26S proteosome. The data presented are difficult to interpret since it is unclear whether the proteosome mechanism explains most or all of PIKK species, as a statistically vali quantitative analysis is not presented. This leaves open the possibility that the proteosome mechanism is present in addition to the Hsp90 mechanism.*

We thank the reviewer for this comment. We agree that we cannot completely exclude the possibility that *Tb*TEL2 can also function as an Hsp90 co-chaperone to help maintain the stability of most PIKK proteins. We have acknowledged this limitation in the Discussion section (page 15, lines 475 – 477). We have also clearly stated that "It is also apparent that *Tb*TEL2 uses different mechanisms to help maintain the *Tb*TOR3 protein level" (Page 15, lines 479 – 480). However, we now have more evidence suggesting that *Tb*TEL2 is unlikely an HSP90 co-chaperone, or at least *Tb*TEL2 is unlikely to do so as a direct PIH1-interacting factor (Fig. S1E, page 13, lines 389 – 398). First, higher eukaryotic TEL2 homologs interact with TTI1 and TTI2 to form the TTT complex, which interacts with the R2TP complex to assume their function as an HSP90 co-chaperone. We have not identified any TTI homologs in *T. brucei* based on sequence homology, suggesting that *T. brucei* may not have a TTT complex or this complex may have TTI subunits that are significantly different from their homologs in higher eukaryotes. We have now discussed this in the Discussion section (page 15, lines 465 – 472). Second, it has been shown that phosphorylation of human TEL2 at S487 and S491 by CK2 is required for its direct interaction with R2TP subunit PIH1D so that it can act as a co-chaperone of HSP90. With the guidance from published sequence alignment (Horejsi et al. 2010 *Mol. Cell*), we confirmed that these CK2 phosphorylation sites are conserved in TEL2s in most higher eukaryotes. The sequence alignment of TEL2 homologs in this region is now shown in Fig. S1E. We found that the serine residues in this region are lost in kinetoplastid TEL2s, even though the surrounding L and D residues are still conserved. This observation further suggests that *Tb*TEL2 does not interact with R2TP to act as a co-chaperone of HSP90. This part of data is described on page 13, lines 389 – 398, and we have added more discussion about this in the Discussion section (page 15, lines 472 – 477).

*2.Part of this reviewer's skepticism is that the structurally similar TEL2 of budding yeast associates with the Tel2-Tti1-Tti2 complex (Hoffmanet al 2016). This raises the obvious question as to whether the T. brucei protein can also form this complex given the level of structural conservation. For a definitive conclusion, this possibility needs to be investigated directly. Alternatively, the authors can limit their conclusion that the TbTel2-mediated PIKK stability is solely mediated through a proteasomal mechanism.*

We thank the reviewer for this comment. We would like to clarify that we never claimed that "the *Tb*TEL2-mediated PIKK stability is solely mediated through a proteasomal mechanism". We merely stated that our observation is **different** from what has been reported for mammalian TEL2, in the Takai et al. 2007. *Cell* paper, where the authors also treated the cells with MG-132, but this did not rescue PIKK protein levels when TEL2 was deleted. To be clear, we have now added that we cannot completely exclude the possibility that *Tb*TEL2 can also function as an Hsp90 co-chaperone to help maintain the stability of most PIKK proteins (page 15, lines 475 – 477).
We have now explored more whether *T. brucei* may also have a conserved TTT complex. Using hTTI1 as query, we did not identify any similar protein by BLASTP. Using ScTTI1 as query, we identified one protein with an E score of 2.7. Similarly, using hTTI2 or ScTTI2 as query, no protein has been identified in the *T. brucei* genome with BLASTP search. This suggests that the *T. brucei* TTT complex, if it exists, is not highly conserved as those in higher eukaryotes. We have described this finding on page 15, lines 465 – 466. This is not surprising for *T. brucei*, an early-branching protist. For example, the *T. brucei* ORC complex that is essential for DNA replication initiation has four kinetoplastid-specific subunits plus *Tb*ORC1 as an ORC1/ORC6 homolog and *Tb*ORC4 as a remote ORC4 homolog. We have discussed this on page 15, lines 466 – 472. These kinetoplastid-specific ORC subunits were only identified when IP-mass spec was performed. In the future, IP *Tb*TEL2 protein complex followed by mass spec analysis will help identify its interacting factors.

*3.Given their hypothesis the authors need to discuss how they envision such a structurally similar protein to be mediated by a distinct mechanism. Do the authors consider this to be a separate kinetoplast branch of function?*

As discussed above, *T. brucei* is an early-branching protist. Even *Tb*TEL2 can form a TTT complex with a similar function as those in higher eukaryotes, it is still possible that the TTI homologs in *T. brucei* are not identifiable based solely on sequence homology. We have now added this in the Discussion section to show that this is not uncommon in *T. brucei* (page 15, lines 466 – 472).

*Is there a DDR in PIKK depleted cells?*
*The authors claim that the absence of sustained gamma H2AX indicates the absence of a DNA damage response pathway. At this same time, they indicate that histone kinases are depleted after TbTEL2 inhibition. If this is the case, how can the absence of the signal indicate the absence of a DDR. This is a point of confusion that needs to be addressed.*

We thank the reviewer for pointing this out. We do not intend to claim that the DDR is absent in *Tb*TEL2 depleted cells. The same pathway should still be there, but since the *Tb*ATR protein level, and presumably the *Tb*ATM protein level too, decreased, the $\gamma$H2A level may not be kept

high in the presence of DNA damage. We observed a similar pattern in the change of the *Tb*RAD51 protein level (added as Fig. S5D), even though both LMPCR (Fig. 4) and PFGE (added as Fig. S5E) showed much more DNA damage in the genome upon *Tb*TEL2 depletion. In addition, we have now added that *T. brucei* has an *Tb*ATM/*Tb*ATR-independent and KKIT5-dependent DNA damage response pathway (Zhou et al. 2019. NAR). It is possible that this pathway may be activated in *Tb*TEL2-depleted cells. We have added discussion about this possibility in the Discussion section (page 16, lines 496 – 498).

*Limitations in Sensitivity*

*The telomere data only shows that there are no large-scale changes in telomere dynamics. However, the assays used are not sensitive enough to draw a definitive conclusion. The absence of a means to detect a single telomere in T. brucei by a STELA like assay is a limitation in this regard. However, the possible improvement in this analysis is suggested by the violin plots after overproduction of TbTEL2. There is some indication that the fraction of shortest telomeres is increased. It may well be a mistake to look for changes in the mean distribution. In this regard, is the increased fraction of shorter telomeres reproducible?*

We thank the reviewer for raising this point.

First, telomere Southern is sensitive enough to determine telomere length changes when cells are cultured for a long-term. This method has been routinely used in many previous published papers to examine telomere length. In the current manuscript, Fig. 2F and Fig. S3, A&C all showed that we can determine the telomere growth rate using this approach. In Fig. 2C, we did not observe any discernable telomere length changes because the cells grew for a very short time. However, the reason why we performed telomere Southern in these cells is to see whether there are any dramatic telomere length changes within a few days. If yes, this would suggest a telomere recombination-mediated telomere length change. If no, then telomere recombination-mediated telomere maintenance (an ALT-like pathway) is unlikely to be activated upon *Tb*TEL2 depletion. We have now added explanation of the rationale why this experiment was performed (page 7, lines 187 – 190).

Second, indeed, it has been shown that in yeast and mammals the shortest telomeres are preferably elongated by telomerase. In *T. brucei*, it has been observed that in telomere healing experiments, newly formed, active *VSG*-adjacent telomere is elongated at a much higher rate than other telomeres. To confirm this is true for all telomeres, including both active and silent telomeres, we have now added more telomere Southern results (Fig. S3). We analyzed telomere growth rates of different telomere fragments first in WT cells. Telomere Southern showed that shorter telomere fragments grow faster than longer telomeres (Fig. S3, A&B, page 7, lines 192 – 207). Subsequently, we repeated this analysis in GFP-*Tb*TEL2 cells. We have now clearly shown that the GFP-*Tb*TEL2 C1 cells expressed GFP-*Tb*TEL2 for an extended period, but C2 cells did not (Fig. 2D). In both GFP-*Tb*TEL2 clones, regardless of whether the cells were cultured with or without Dox, we did not see any significant difference in the pattern of how telomeres grow (Fig. 2, F&H; Fig. S3, C&D; page 8, lines 209 – 232). Therefore, we conclude that perturbing *Tb*TEL2's expression level does not affect the telomere growth rate.

*The IP studies rule out, as the authors point out, a stable association of TbTEL2 with other telomeric proteins. However, in context of chromatin, this is a rather insensitive assay. The authors should reproduce their PICH assay with the overproduced tagged TEL2 species. This would be a more critical test of the association with Tel2 with other telomere proteins.*

*The association of TbTEL2 by IP with ATR is important. It is unfortunate that ATM association could not be tested. Have the authors tried FLAG tagging of ATM. This has often led to the production of active protein. Could the authors state the technical limitation of tagging TEL2 in Tb. Given that Tel2 has been demonstrated to associate with ATM in yeast, this is an important omission.*

We thank the reviewer for raising this point. To introduce what is known about TEL2 homologs more clearly, we have now added that none of the known TEL2 homologs have been shown to be a core telomere protein component (page 4, lines 91 – 93; page 14, lines 424 - 426). We have now also added the observation from tryptag.org, where *Tb*TEL2 is mostly localized in the cytoplasm (page 9, lines 247 – 250). Although *Tb*TEL2 was identified in our PICh experiment, only two peptides were identified. Together with other results, we conclude that *Tb*TEL2 is not a **core** component of the telomere complex, and we also acknowledge that *Tb*TEL2 may transiently associate with the telomere chromatin (page 14, lines 427 – 429).
We tried *Tb*ATM tagging for more than two years without success. Tagging an endogenous *TbATM* allele was successful in procyclic *T. brucei* cells, but it has not been reported in bloodstream form cells. In addition, since *TbATM* is a very big gene, expressing a tagged ectopic *TbATM* allele is not trivial.
We do not know whether the ectopic *Tb*TEL2 is overexpressed. Without the *Tb*TEL2 antibody, it would be very hard for us to estimate the *Tb*TEL2 expression level relative to the endogenous protein. This is why we only stated "expressing an ectopic *TbTEL2* allele" rather than "*Tb*TEL2 overexpression".

*Interpretation of LMPCR*
*Some verification of the presence of DNA breaks would be preferable given the heterogenous set of breaks produced by the I LMPCR assay. As a control, conduct an experiment in which the end structure is resected making breaks unable to ligate to ensure the absence of any species*
*Another approach to test for the validity of the LMPCR is using CHEF gene analysis to detect breaks directly (as well as other forms of recombination that is occurring).*

We thank the reviewer for this suggestion. LMPCR has been used to detect subtelomeric DNA breaks in *T. brucei* previously by our and other labs (Boothroyd et al. 2009. Nature; Jehi et al. 2014, Cell Res.; Jehi et al. 2016. PLoS One; Glover et al. 2013. PLoS Pathogen). We are using the same protocols and have included necessary controls. To further verify that DNA breaks occur in *Tb*TEL2-depleted cells, we have now performed PFGE of undigested genomic DNA from *Tb*TEL2 RNAi cells before and after depleting *Tb*TEL2. The result is shown in Fig. S5E. Indeed, we see that gDNA was much more smeared after *Tb*TEL2 is depleted 48 and 72 hrs.

***Textual Issues***
*Line 75. Explain why an analysis of telomeres is central to understanding VSG switching. This is a critical issue that is omitted in the Introduction.*

We thank the reviewer for pointing this out. We have now added that telomere length can also affect VSG switching rate (page 4, lines 78 – 80). In combination of other reasons we have listed – locations of *VSG* genes, telomere proteins as key regulators of antigenic variation – we hope we have explained the rationales of our study more clearly.

Line 261: The authors demonstrate in Figure 3 the presence of other classes of genetic change but do not present any primary data substantiating these rearrangements. How were the different classes defined? There is also no statistical analysis of the differences in the presence and absence of Tel2.

We thank the reviewer for this comment. First, we realized that in our original assay, the S/ev cell yielded a small number of switchers (which is expected as the VSG switching rate in these cells are low), so we performed new VSG switching assays in the S/ev cells and obtained more switchers. This allowed us to recover more switchers from the control cells so that we identified switchers using different switching mechanisms. Second, we have now added Table S5 that lists phenotypes and genotypes of all switchers we obtained from S/*Tb*TEL2-SKOi and S/ev cells. In addition, the VSG switching assay is now described with more details (pages 9 – 10, lines 278 – 288; pages 17 – 18, lines 539 – 558), and the VSG switching assay principle is now illustrated in Fig. S5A. We have also added a statistical analysis to compare the VSG switching mechanism distributions in S/*Tb*TEL2-SKOi and S/ev, which is described on page 10, lines 290 – 295 and page 20, lines 638 - 645.

*Line 318: The level of each transcript is given in Figure 5. However, what were the number of repetitions and variations between repeats?*

We thank the reviewer for this comment. We have now added Table S4 listing raw data for all qRT-PCR experiments. In Fig. 5F, we realize that we did not include *Tb*ATM data initially. Now we have added this result. Individual experiment data points were plotted. All PIKK genes were analyzed for at least 5 times, and *Tb*TOR1 was analyzed 7 times. Control genes were analyzed 2-3 times. For each data point, mean and standard deviation are indicated by bars. This is now described in the figure legend of Fig. 5F.

*Line 323. The absence of a major shift in RNAs show that PIKK levels are maintained by a post-transcriptional mechanism (not post-translational) as stated. Although unlikely, the authors do not discuss the possibility of reason for ruling out a translational mechanism.*

We thank the reviewer for pointing this out. We have revised the term to “post-transcriptional” (page 12, line 364). Figure 7 shows that for *Tb*ATR, *Tb*TOR1, *Tb*TOR2, and *Tb*TOR4, adding MG-132 rescued these proteins to their WT level (or higher) even after *Tb*TEL2 was depleted, suggesting that for these proteins, blocking 26S proteasome-mediated degradation is a major mechanism how *Tb*TEL2 helps maintain their stability. As for *Tb*TOR3, we have stated that other mechanism(s) is involved. Without any data, we simply did not speculate that translation regulation is involved, although we did not exclude this possibility either.

Line 342. The authors show that in all cases except for TOR3, MG132 reverses the depletion. However, these analyses seem to be qualitative so that it is difficult to ascertain whether the reversal accounts for all or part of the TbTEL2-mediated PIKK loss of stability.

We thank the reviewer for this comment. Western blots indicated MG-132 treatment rescued these proteins back to WT level or higher, although we agree that this assay is not very quantitative. Nevertheless, we did not mean to claim that protecting PIKK from proteasome-mediated degradation is the sole mechanism of *Tb*TEL2's function in maintaining PIKK protein levels. As explained above, we have now added that we cannot completely exclude the possibility that *Tb*TEL2 can also function as an Hsp90 co-chaperone to help maintain the stability of most PIKK proteins (page 15, lines 475 – 477). In addition, we added more sequence alignment (Fig. S1E) that strongly suggests that *Tb*TEL2 does not interact directly with PIH1 to act as a co-chaperone of HSP90. This part of data is described on page 13, lines 389 – 398, and we have added more discussion about this in the Discussion section (page 15, lines 472 – 477).

*Line 363: Although the authors show the kinetoplastid TEL2 homologs are most conserved, the functional significance of this is unclear since this represents the first functional analysis.*

We thank the reviewer for this suggestion. We have added one more paragraph at the end of the Discussion section to better state the significance of this study (page 16, lines 508 – 516).

*Line 392. Please explain types of DDR pathways independent of gamma H2A.*

We thank the reviewer for this comment. We have now added that a kinetochore dependent DDR pathway is not ATM/ATR dependent (Zhou et al. 2019. NAR). It is possible that this pathway may be activated in *Tb*TEL2-depleted cells. We have added discussion about this possibility in the Discussion section (page 16, lines 496 – 498).

*Line 460: VSG Switching Assay: The description of this assay In the Materials and Methods does not seem to match that in the text. The text states that selection for VSG2 is released and switching is analyzed. Please clarify.*

We thank the viewer for pointing this out. The VSG switching assay is now described with more details (pages 9 – 10, lines 278 – 288; pages 17 – 18, lines 539 – 558), and the VSG switching assay principle is now illustrated in Fig. S5A. Raw data from this assay have been added in Table S5.

Line 474: What do you mean by "VSG2 rabbit antibody (without the cross-reaction proportion)"?

We have revised this to "VSG2-specific antibody" (page 9, line 283).

***Figure Related Issues***

*Figure 1:*

*A.Provide a brief description of the functional significance of the TEL2 arm and telomere length regulation protein motifs.*

We have added more explanation of the two Pfams in Figure 1 legend.

*B.Explain the meaning of the color coded LDDT values.*

We have added the meaning of LDDT in Figure 1 legend.

C-E, provide statistical power of comparisons associated with each of these comparisons.

We use ChimeraX to align these structures. We have now added more alignment parameters in Table S2.

*Figure 2(all). Please present panels in order of presentation*

We have tried to arrange the panels in their natural order now.

*Figure 2F.*
*Are the species denoted by the arrows based on a previous analysis that verified their identity of single species increasing in size?*

*T. brucei* has nearly 250 telomeres. In clonal population, such as recently established strains, multiple different telomere bands can be detected when a telomeric probe is used in Southern hybridization. Exactly how many telomeres each band represents is unknown and will be different from clone to clone. However, we can clearly identify a subset of prominent telomere bands and follow their size changes. We only picked clear prominent telomere bands for quantification.

*Figure 2G.*
*Is the lower bound of the GFP-TbTel2 reproducible in violin plots? How much overproduction is achieved with the GFP proteins?*

We cannot determine whether GFP-*Tb*TEL2 is overexpressed as we do not have *Tb*TEL2 antibody. That's why we only described this as "expressing an ectopic *TbTEL2* allele". We have now quantified more telomere fragment growth rates and did additional statistical analyses to show that shorter telomere fragments are elongated faster and that expressing an ectopic *TbTEL2* allele does not change the telomere elongation pattern (pages 7 – 8, lines 177 – 232).

*Figure 3 (all) Place the panels in order of presentation*

We have rearranged the panels in their natural order.

*Figure 3A> The error bars for fold changes in RNA levels are quite large. What is the reason for this?*

We have performed this experiment with several different biological repeats, each time cells are induced differently.

Figure 6. Present the aggregate data for all the repeats and quantification of data.

All raw quantification data are now reported in Table S6.

*Supplementary Figure 1*
*The legends indicates that the sequences were compared to the consensus sequence. There is no TEL2 consensus. Please explain.*

We thank the reviewer for pointing this out. We have now shown the consensus sequence.

*Supplementary Figure 4*
*What do the numbers above each lane refer to?*

These are different clones. We have added an explanation in the figure legend.

*The myc tagged species appear to be at the limit mobility of the gel in A, and E. A better separation is needed for the identification.*

The PIKKs are large proteins. The current protein electrophoresis already allows us to detect these tagged proteins.

*Reviewer #3 (Comments for the Author):*

*This manuscript by Sayeed et al. identifies the Tel2 protein of T. brucei and begins to characterize some of its functions. The manuscript is clearly written and straightforward with the results shown justifying the conclusions drawn. The work described is a modest but important step forward in the field. I have only minimal criticisms, listed below.*

*Is there data on whether the GFB-TEL2 fusion is functional, having the essential TEL2 function? Or, is this just assumed in the ectopic expression work? Does the level of expression of the fusion protein change over the growth time course? This might be worth knowing given the bit of change in growth occurring early on.*

We thank the reviewer for this comment. Adding an N-terminal F2H to *Tb*TEL2 does not affect *Tb*TEL2's essential functions (Fig. S2D), so it is expected that GFP-*Tb*TEL2 still retains its

essential functions. We have checked the GFP-*Tb*TEL2 expression after ~ 120 PDs of growth in the two clones we obtained. C1 still expressed GFP-*Tb*TEL2 but C2 lost the expression. We have added this data to Fig. 2D. Telomere length changes were examined in both C1 and C2, where C2 was used as a control. Quantifications of both telomere Southern gels are now shown in Fig. 2 and Fig. S3.

Re: Spectrum03516-25R1 (***Trypanosoma brucei* TEL2 inhibits VSG switching and protects PIKKs from the 26S proteasome-mediated degradation**)

Dear Dr. Bibo Li:

I'm pleased to inform you that your manuscript has been accepted, and I am forwarding it to the ASM production staff for publication. Your paper will first be checked to make sure all elements meet the technical requirements. ASM staff will contact you if anything needs to be revised before copyediting and production can begin. Otherwise, you will be notified when your proofs are ready to be viewed.

**Data Availability:** ASM policy requires that data be available to the public upon online posting of the article, so please verify all links to sequence records, if present, and make sure that each number retrieves the full record of the data. If a new accession number is not linked or a link is broken, provide production staff with the correct URL for the record. If the accession numbers for new data are not publicly accessible before the expected online posting of the article, publication may be delayed; please contact ASM production staff immediately with the expected release date.

**Publication Fees:** For information on publication fees and which article types have charges, please visit our website. We have partnered with Copyright Clearance Center (CCC) to collect author charges. If fees apply to your paper, you will receive a message from no-reply@copyright.com with further instructions. For questions related to paying charges through RightsLink, please contact CCC at ASM_Support@copyright.com or toll free at +1-877-622-5543. CCC makes every attempt to respond to all emails within 24 hours.

**ASM Membership:** Corresponding authors may join or renew ASM membership to obtain discounts on publication fees. Need to upgrade your membership level? Please contact Customer Service at Service@asmusa.org.

**Embargo Policy:** A press release may be issued as soon as the manuscript is posted on the Spectrum Latest Articles webpage. The corresponding author will receive an email with the subject line "ASM Journals Author Services Notification" when the article is available online.

The ASM Journals program strives for constant improvement in our submission and publication process. Please tell us how we can improve your experience by taking this quick Author Survey.

Thank you for submitting your paper to Spectrum.

Sincerely,
Galadriel Hovel-Miner
Editor
Microbiology Spectrum

Reviewer #2 (Comments for the Author):

The authors have done an excellent job in addressing my concerns.
